# A small bioactive glycoside inhibits epsilon toxin and prevents cell death

**DOI:** 10.1242/dmm.040410

**Published:** 2019-10-01

**Authors:** Abhishek Shivappagowdar, Soumya Pati, Chintam Narayana, Rajagopal Ayana, Himani Kaushik, Raj Sah, Swati Garg, Ashish Khanna, Jyoti Kumari, Lalit Garg, Ram Sagar, Shailja Singh

**Affiliations:** 1 Department of Life Sciences, School of Natural Sciences, Shiv Nadar University, Greater Noida, Uttar Pradesh 201314, India; 2 Department of Chemistry, School of Natural Sciences, Shiv Nadar University, Greater Noida, Uttar Pradesh 201314, India; 3 Gene Regulation Laboratory, National Institute of Immunology, New Delhi 110067, India; 4 Special Centre for Molecular Medicine, Jawaharlal Nehru University, New Delhi 110067, India; 5 Department of Chemistry, Institute of Science, Banaras Hindu University, Varanasi 221005, India

**Keywords:** β-PFT, Glycoside-4, Structure-activity relationship, Oligomerization, Micelle formation

## Abstract

*Clostridium perfringens* epsilon toxin (Etx) is categorized as the third most lethal bioterrorism agent by the Centers for Disease Control and Prevention (CDC), with no therapeutic counter measures available for humans. Here, we have developed a high-affinity inhibitory compound by synthesizing and evaluating the structure activity relationship (SAR) of a library of diverse glycosides (numbered 1-12). SAR of glycoside-Etx heptamers revealed exceptionally strong H-bond interactions of glycoside-4 with a druggable pocket in the oligomerization and β-hairpin region of Etx. Analysis of its structure suggested that glycoside-4 might self-aggregate to form a robust micelle-like supra-molecular complex due to its linear side-chain architecture, which was authenticated by fluorescence spectroscopy. Further, this micelle hinders the Etx monomer-monomer interaction required for oligomerization, validated by both surface plasmon resonance (SPR) and immunoblotting. This phenomenon in turn leads to blockage of pore formation. Downstream evaluation revealed that glycoside-4 effectively blocked cell death of Etx-treated cultured primary cells and maintained cellular homeostasis via disrupting oligomerization, blocking pore formation, restoring calcium homeostasis, stabilizing the mitochondrial membrane and impairing high mobility group box 1 (HMGB1) translocation from nucleus to cytoplasm. Furthermore, a single dosage of glycoside-4 protected the Etx-challenged mice and restored normal function to multiple organs. This work reports for the first time a potent, nontoxic glycoside with strong ability to occlude toxin lethality, representing it as a bio-arm therapeutic against Etx-based biological threat.

## Abstract (precis)

**Summary:** The authors developed a potent, glycoside-based inhibitor with strong ability to occlude epsilon toxin lethality both *in vitro* and *in vivo*.

## INTRODUCTION

Microorganism-based toxins as bio-arms are a potential threat to humans owing to their ease of availability and low-cost production. Among the enlisted category of bioweapons by the Centers for Disease Control and Prevention (CDC) (CDC, 2000), *Clostridium perfringens* epsilon toxin (Etx) has been categorized as the third most potent toxin after botulinum neurotoxin (BoNT) and anthrax, and is a classified type B agent. Out of five strains (A-E) of *C. perfringens*, epsilon toxin is secreted by toxinotypes B and D. It causes fatal enterotoxaemia, also called the kidney pulp disease, in domestic ruminants, resulting in heavy economic losses (Songer, 1996; Titball, 2009). Apart from kidneys, this toxin also affects the brain by enhancing permeability of the blood-brain barrier (Morgan et al., 1975). If an Etx-based biological attack was to occur, the possible exposure routes might include inhalation of aerosolized particles and contamination via food and water, leading to multiple organ failure and high morbidity in humans. Despite its remarkable structural similarity with the crystal structure of aerolysin toxin, the degree of lethality by Etx seems to be 100-fold higher than aerolysin (Cole et al., 2004; Minami et al., 1997). This significant difference in its activity is attributed to its specific amino acid composition. Etx belongs to category of beta-pore-forming toxins (β-PFTs) (Knapp et al., 2009). The suggestive roles for its three domains explain its structure-activity dynamics, including; receptor binding (domain I; N-terminus); hairpin insertion plus channel formation (domain II; central region); and homo-oligomer formation and proteolytic activation (domain III; C-terminus). Addition of purified toxin causes rapid efflux of K^+^ and an increase in intracellular Cl^−^ and Na^+^, with marked swelling, mitochondrial disappearance, and membrane blebbing and disruption, leading to cell death (Chassin et al., 2007; Lindsay, 1996; Shortt et al., 2000). Additionally, few cultured animal cell lines are sensitive to the toxin, for example Madin-Darby canine kidney (MDCK), mpkCCD_C14_ and G-402, and can represent the toxin-mediated cellular phenotypes (Fernandez Miyakawa et al., 2011; Shortt et al., 2000). As per previous reports (Knapp et al., 2009), domain II is known for playing crucial roles in oligomerization and pore formation, and amino acid mutation at this domain has been implicated in altered cytotoxicity. Based on this information, we hypothesize that accessing the druggability of domain II in Etx might lead to the development of a promising antidote against epsilon toxin. To antagonize Etx, various approaches, including the use of antibodies (McClain and Cover, 2007), formalin-inactivated vaccine, anti-sera, an equine-derived antitoxin and three small-molecule inhibitors (Lewis et al., 2010), have been developed so far. None of these therapeutic measures are effective in counteracting the effects of the toxin.

To address this issue, using a green synthesis route we have synthesized a library of D-glucose- and D-glucosamine-derived glycosides with a hydrophilic head group and different chain lengths of the tail (lipophilic group). These molecules were found to be completely non-toxic in mammalian cells as well as in animal models. Screening for anti-Etx activity both *in vitro* and *in vivo* reports glycoside-4 as a potential antidote against Etx and can be further developed as a first line of defence against bio-terrorizing agents in humans.

## RESULTS

### Synthesis of glycoside-based compounds using a green synthetic route

The glycoside-derived compounds were designed and synthesized using the commercially available D-glucose-1 and D-glucosamine-2 molecules, which were coupled along with various short- to long-chain alcohols under acidic conditions. The ethyl glycoside D-glucose glycoside-1 was prepared by refluxing in ethanol for 24 h in the presence of amberlite-H^+^ IR-120, which gave us the desired product glycoside-1 as an anomeric mixture in good yield as described in the scheme for synthesis (Fig. S1A). This synthesis followed a green route to prepare desired glycoside as it does not involve any further purification and amberlite resin can be reused by activating it with 1 N HCl in MeOH. Similarly, other compounds in this series (glycoside-2 to glycoside-6) were prepared by adopting similar reaction protocol (see Materials and Methods), which resulted in moderate to good yields (Fig. S1A). The alkyl glycosides of D-glucosamine glycoside-7 to glycoside-12 were prepared by refluxing D-glucosamine-2 with corresponding alkyl alcohols for 24 h in the presence of amberlite-H^+^ IR-120 resin, which gave the desired products as an anomeric mixture in good yield as shown (Fig. S1B). This synthesis was simple: no further workup was required and the used amberlite resin was reusable after activating it with 1 N HCl in MeOH. Further characterizations of all 12 compounds (Table S1) were done using nuclear magnetic resonance (NMR) (supplementary text).

### Molecular dynamics simulation and *in silico* docking revealed a unique, druggable pocket in the Etx heptamer

**Fig. 1.**
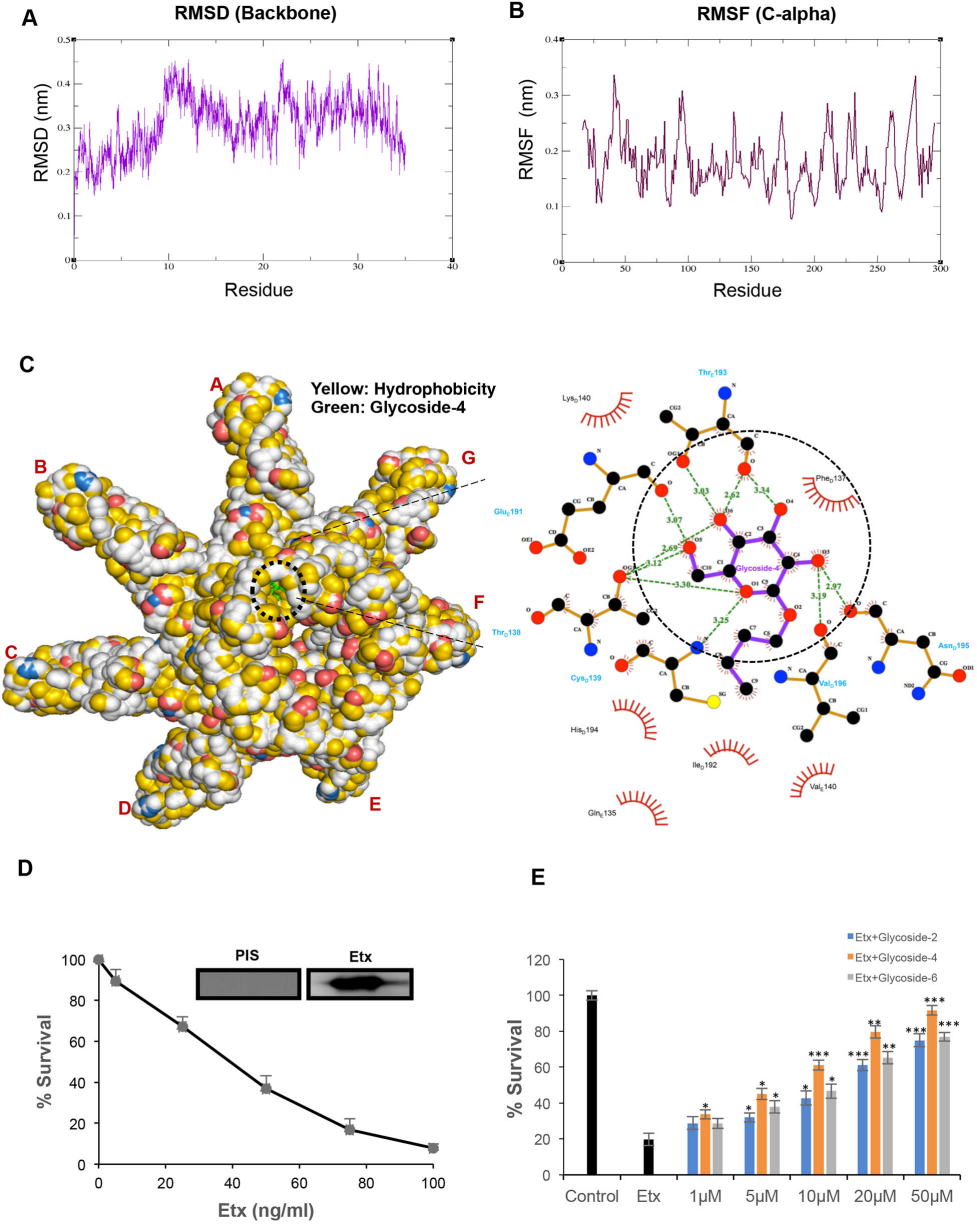
***In vitro* toxicity screening and** ***in silico* docking reveal glycoside-4 as the lead compound.** (A) RMSD curve of heptameric Etx showed minimal changes in stability post-MD simulation run of 40 ns. (B) RMSF curve corroborated the RMSD curve with no severe changes in RMSF during the MD run. (C) Heptameric surface model of ligand-bound Etx denoted by the degree of hydrophobicity and the surface accessible for ligand binding using a YRB scheme. The defined 3D virtual pocket has been highlighted and was found to have hydrophobic patches. Ligplot interaction analysis of the lead compound (glycoside-4) with Etx revealed ten H-bonds within the defined hydrophobic pocket. (D) Etx was purified and confirmed by western blotting. Antibody generated in mice specifically reacted and a single band was detected (inset). MDCK cells were challenged with the indicated (5-100 ng/ml) concentration of Etx, the cells were incubated for 1 h and survival was affirmed by MTT assay. PIS, pre-immune sera. (E) Different concentrations of glycosides (2, 4, 6) were screened against Etx-treated MDCK cells and the ability to rescue was determined using MTT assay (***P<0.005, **P<0.01, *P<0.05, Student's *t*-test). Error bar represents mean±s.d.

The crystal structure of *C. perfringens* Etx was obtained from the Protein Data Bank (PDB) database (ID: 1UYJ; www.rcsb.org). Since the crystal structure of the Etx pre-pore was unavailable, we constructed the heptameric pre-pore form, exactly mimicking its active and pre-pore-forming state. Since the inactive protoxin has to be activated by proteolytic removal of the 11-13 and 22-29 residues from the N- and C-terminus, respectively (Popoff, 2011), we have removed the same stretch of residues from the whole protein. To emphasize, the cleavage at the C-terminus is particularly important for the biological activity and the ability to form large sodium dodecyl sulfate (SDS)-resistant heptameric complex (Miyata et al., 2001). To elucidate the stability of the large heptameric model of Etx, we have performed molecular dynamics (MD) simulation. The energy coordinates were observed to converge after 4000 steps, at which point the lowest potential energy (−71,271,584 kJ mol^−1^) was calculated. The root-mean-square distance (RMSD) and root-mean-square fluctuation (RMSF) curves were found to be devoid of any severe fluctuations, representing a stable heptamer (Fig. 1A,B and Fig. S2A). The intra-molecular bonding and hydrophobicity analysis of this Etx heptamer revealed predominant existence of amphipathic residues constituting a druggable pocket within domain II (Fig. 1C), which is involved in oligomerization and membrane insertion. Initially, we performed *in silico* docking of 12 glycoside derivatives with the Etx monomer (PDB: 1UYJ), to understand whether these compounds could bind to the monomeric form as well. The result demonstrated relatively weaker binding (−4.1 to −4.8 kcal/mol) within the pocket (Table 1 and Fig. S2B). Following this, we performed *in silico* docking of 12 glycoside derivatives with the Etx heptamer. The docked heptameric Etx-ligand complexes revealed stronger binding energies (−5.8 to −7.0 kcal/mol) localized within this pocket (Table 1 and Fig. S2C) as compared to the monomeric Etx-ligand (Table 1 and Fig. S2B).

**Table 1.**
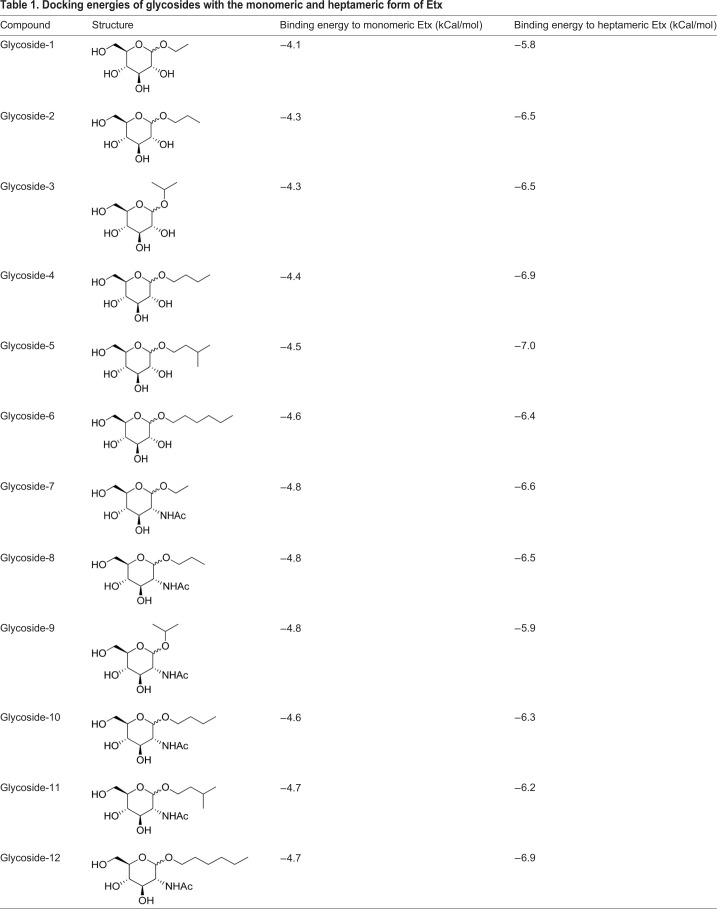
**Docking energies of glycosides with the monomeric and heptameric form of Etx**

### *In vitro* toxicity screening of all glycoside derivatives against Etx shows glycoside-4 as the lead compound

We evaluated the cytotoxicity of the 12 glycoside-based molecules in mammalian cell lines HepG2 and MDCK, which showed no toxicity even at higher (100 µM) concentrations (Fig. S2D,E). Since MDCK is highly sensitive to Etx, we have evaluated the impact of all 12 glycoside derivatives for anti-toxin activity in Etx-challenged MDCK cells by using the mitochondrial membrane potential (ΨΔm) indicator MTT [3-(4,5-dimethylthiazol-2-yl), 2,5 diphenyltetrazolium bromide] (Lindsay, 1996; Shortt et al., 2000). To achieve this, we purified biologically active Etx according to a previous report from our group (Goswami et al., 1996) and activated it using trypsin (Hunter et al., 1992). We immunized BALB/c mice with 10 µg activated Etx and collected the serum. The antisera against the protein was able to detect the single and specific band corresponding to the expected size (∼31 kDa) of purified Etx. Pre-immune sera (PIS) was used as a control and did not react with purified Etx (Fig. 1D). To further authenticate the activity of Etx, we monitored the cytotoxicity by using MTT (Lindsay, 1996; Shortt et al., 2000). The metabolic viability of MDCK cells was found to be significantly decreased with increasing concentration of Etx treatment. The IC_50_ of the purified Etx was validated to be 50 ng/ml, whereas 100 ng/ml toxin was enough to kill all the cells (Fig. 1D). Untreated cells showed 100% cell survival. Since Etx at a concentration of 100 ng/ml could impose maximum toxicity, we have further used this concentration for all *in vitro* analyses. Initially, all the glycosides were screened at 20 µM and 50 µM concentration, in which three (glycoside-2, -4 and -6) showed greater than 50% survival compared to Etx (Fig. S3A). Further, all three glycosides showed concentration-dependent protection of Etx-treated MDCK cells. Glycoside-4 was found to be the lead molecule among them, with highest survival efficiency (Fig. 1E). Thus, it was used for both *in vitro* and *in vivo* assays to explore its protective effect against Etx lethality.

### Structural analysis of glycoside-4 interaction with the Etx heptamer exposed the interacting residues within the druggable pocket of Etx

To understand the structure-activity relationship of glycoside-4, we performed docking of glycoside-4 to the annotated amphipathic pocket present in domain II of Etx (Fig. 1C and Fig. S3B). The results demonstrated a total of ten strong intermolecular H-bonds between the pocket residues and glycoside-4. The threonine residues at 138 and 193 could make three H-bonds each, while Glu191, Cys139, Asn195 and Val196 made a total of four H-bonds with glycoside-4 (one each) (Fig. 1C). This cluster of interacting residues was found to be restricted to the β-hairpin structure (Fig. S3B). Upon optimization of the 3D grid parameters, the lead compound, glycoside-4, showed higher binding affinity (−6.9 kCal/mol) and its best docked conformation was further analyzed for polar contacts, including salt bridges and H-bonding (Tables S2 and S3). The results demonstrated ten intermolecular H-bonds (2.6-3.1 Å) and few intramolecular salt bridges within the defined pocket of ligand-bound complex (Fig. 1C and Tables S2 and S3). In contrast, we obtained only one H-bonding when we docked the Etx monomer to glycoside-4 (Fig. S2B). We assume that strong binding of glycoside-4 within the domain II region of Etx monomers in the heptamer might introduce steric hindrance and might block the process of the oligomerization, leading to destabilization of β-barrel insertion.

### Mechanistic insights revealed that glycoside-4 could drastically disrupt oligomerization, block pore formation and impair calcium influx

Since the heptameric pre-pore complex of Etx employs the β-barrel hairpin structure to make pores in the plasma membrane (Knapp et al., 2009), we hypothesize that glycoside-4 could alter the membrane insertion by binding to critical residues in the pre-pore structure. To address this issue, we have characterized the morphometric alteration and membrane-destabilizing effects of Etx in MDCK cells. In accordance with an earlier report (Petit et al., 1997), Etx-exposed MDCK cells showed rapid changes in cell morphology and membrane architecture in a dose-dependent manner (Fig. S4A). These morphological changes are often associated with alterations in the cell architecture such as cell-cell and cell–extracellular-matrix (ECM) interactions that determine the barrier integrity, measured by transepithelial electric resistance (TEER) (Stolwijk et al., 2015). To assess whether Etx causes a loss in the TEER, we added Etx to a confluent monolayer of MDCK cells grown in eight-well chambers for electrical cell impedance sensing (ECIS)-based analysis. A rapid decrease in TEER was observed, with maximum changes in the first 2 h of Etx addition, which complemented our metabolic viability data (Fig. S4B and Fig. 1D). No changes in TEER could be observed in the control cells (Fig. S4B). Further, to observe the membrane changes in real time, we performed live-cell video microscopy. The cells displayed prominent swellings followed by membrane blebbing. Subsequently, the blebs grew bigger (Fig. S4C, Movie 1), validating the cellular intoxication imposed by purified Etx used in our study. Since structural insights indicated strong binding of glycoside-4 to the annotated region for oligomerization and membrane insertion in the druggable pocket of Etx, we hypothesized that glycoside-4 might block the oligomerization and/or insertion *in vitro*.

**Fig. 2.**
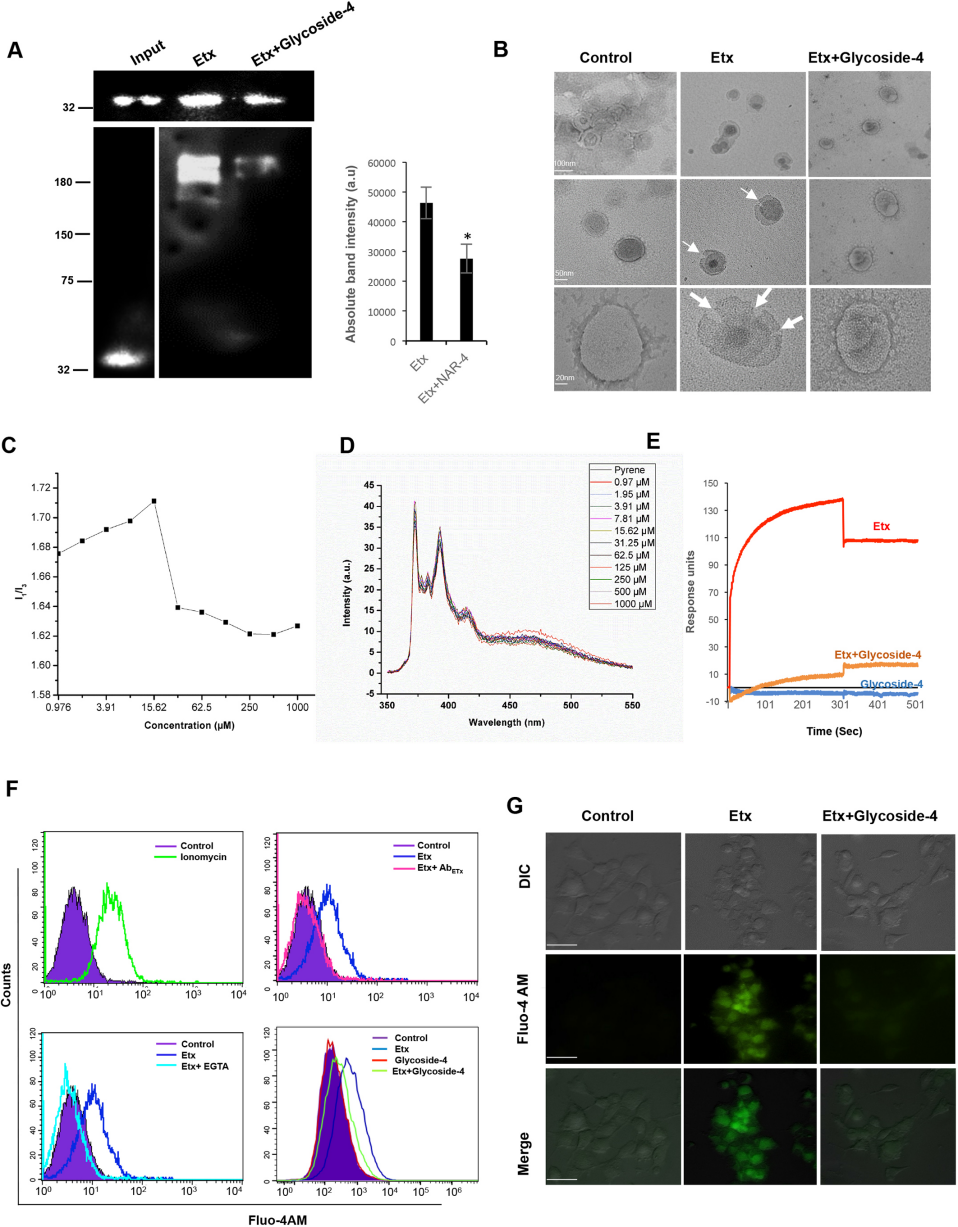
**Glycoside-4 impairs oligomerization and pore formation in Etx-treated MDCK cells.** (A) The ability of Etx to bind to the MDCK cells was observed in the presence or absence of glycoside-4 by western blot (top panel). No difference in binding between the Etx- and glycoside-4-treated cells was observed. MDCK cells were treated with Etx or along with glycoside-4 and the ability to form high-molecular-mass oligomers was observed by western blot (bottom panel). A decrease in oligomerization in glycoside-4-treated cells could be seen. Absolute band intensities for Etx and with glycoside-4 are shown (*P<0.05, Student's *t*-test). Error bar represents mean±s.d. (B) Uni- and multi-lammellar lipid mixtures (50% cholesterol+50% DPPC) were treated with the Etx alone or with glycoside-4. TEM images show reduced pore formation on the lipid surface in the presence of glycoside-4 compared to the control liposomes. Scale bars are represented in the figure. (C) Changes in the ratio of pyrene (1.0×10^−6^ M) vibrational band intensities (*I*_1_/*I*_3_) as a function of different concentrations (0.97-1000 µM) of glycoside-4 in water is plotted. The graph suggests the critical micelle concentration of glycoside-4 in water to be 15.625 µM. (D) Fluorescence spectra of pyrene (1.0×10^−6^ M) in the presence of glycoside-4 at various concentrations (0.97-1000 µM) in water is depicted. (E) Etx was immobilized onto a nickel-charged NTA SPR chip. Etx alone, glycoside-4 alone and Etx in combination with glycoside-4 was injected over immobilized Etx. Interactions between Etx-Etx monomers was drastically reduced in the presence of glycoside-4. (F) Intracellular Ca^2+^ levels were monitored in MDCK cells treated with ionomycin and Fluo-4 AM. Clear intensity shift was observed compared to the control cells. MDCK cells were treated with Etx and anti-Etx antibody for 1 h and treated with Fluo-4 AM. The cells incubated with Etx and antibody mixture showed no Ca^2+^ influx, whereas cells with Etx alone showed drastic Ca^2+^ influx. MDCK cells were treated with Etx in the presence or absence of EGTA for 1 h and Ca^2+^ increase was seen. Prominent Ca^2+^ influx was observed in Etx-treated cells, whereas no influx was observed in cells incubated with EGTA-containing media. MDCK cells were treated with Etx and glycoside-4 to check for Ca^2+^ influx. A decreased influx was observed upon treatment with glycoside-4 compared to with Etx treatment alone. (G) MDCK cells were treated with Etx or in combination with glycoside-4 to check for Ca^2+^ entry into the cells. Fluorescent images show complete reduction in Ca^2+^ entry after treatment with glycoside-4. Scale bars: 20 μm.

To explore this, initially we checked the glycoside-4 activity on binding of Etx monomer (31 kDa) to MDCK cells. To prove this, we treated MDCK cells with Etx alone or in combination with glycoside-4 for 1 h at 4°C, followed by western blotting of the crude MDCK pellet. The results indicated that glycoside-4 had no effect on the binding of Etx monomer to the cell membrane (Fig. 2A, top panel). This coincided with our *in silico* observation, which suggested that glycoside-4 has weaker binding affinity for Etx monomers (Table 1 and Fig. S2B). Next, to evaluate whether glycoside-4 can also impact oligomerization, we incubated the Etx along with MDCK in the presence or absence of glycoside-4 at 37°C for 1 h, as oligomerization occurs at the same temperature *in vitro* (Petit et al., 1997). Following incubation, western blotting was performed, and the immunoblot revealed a substantial decrease in heptamer intensity as compared to the Etx (Fig. 2A, bottom panel). Since oligomerization is the first step towards membrane insertion, followed by pore formation (Miyata et al., 2001), we assumed that disruption of oligomerization by glycoside-4 might lead to blocking of pore formation. To confirm this, we performed transmission electron microscopy (TEM) using lipid vesicles. Upon Etx addition, ‘pizza-slice-shaped tearings’ could be detected in liposomes, whereas, upon incubating glycoside-4 with Etx-treated liposomes, these tearings disappeared, indicating strong disruption in pore formation (Fig. 2B). This result is consistent with our western blot data (Fig. 2A) and indicates that glycoside-4 strongly hinders the process of oligomerization.

It is well established that monosaccharide and disaccharide derivatives are well known to self-assemble, because of having poly-hydroxy groups, and form various complexes in aqueous medium and form aggregates (Sonoda and Skaf, 2007; Vidyasagar et al., 2011). Since glycoside-4 is a carbohydrate-based molecule, we wanted to check for its micelle-forming ability. We performed fluorescent spectroscopy to determine the critical micelle concentration (CMC) in aqueous solution. The pyrene spectrum shows several vibronic peaks, and the intensity ratio of the first (at 373 nm) and third (at 383 nm) vibronic peaks, *I*_1_/*I*_3_, is a sensitive indicator of the polarity of the pyrene microenvironment (Dong and Winnik, 1984; Kalyanasundaram and Thomas, 1977). The first observation of the decrease in the ratio of pyrene vibrational band intensity (*I*_1_/*I*_3_) value demonstrates the onset of the formation of micellar assemblies (Baker et al., 2001; Nakahara et al., 2005) and thus indicates the CMC of glycoside-4 in water to be 15.625 µM (Fig. 2C,D). This value is closely related to the effective inhibition concentration of glycoside-4 (Fig. 1E). Now, to check whether the Etx monomer-monomer interaction required for oligomerization could be inhibited in the presence of glycoside-4, we performed surface plasmon resonance (SPR) at CMC. The Etx was immobilized on the SPR chip and, on passage of Etx, a clear protein-protein interaction could be observed (Fig. 2E). However, upon passage of the mixture containing Etx and glycoside-4, no Etx (protein-protein) interaction could be seen (Fig. 2E), suggesting the attenuation of stable oligomer formation in the presence of glycoside-4.

It is evident that calcium influx is one of the major indicators of Etx-triggered cell death (Chassin et al., 2007). We assumed that disruption of pore formation by glycoside-4 might also block the calcium influx *in vitro*. To have a proof of concept, initially we evaluated the Ca^2+^ ingress in MDCK cells in the presence of Etx by FACS analysis (Fig. 2F, Fig. S4D and Movie 2). The data showed that Etx could induce Ca^2+^ similar to ionomycin, a positive control. Treatment with EGTA, an extracellular Ca^2+^ chelator, blocked the Etx-invoked influx (Fig. 2F). Assuming that glycoside-4 could enforce the blockage of the Etx-triggered Ca^2+^ influx, similar to the EGTA-mediated response in MDCK, we treated the cells with Etx in the presence of glycoside-4 in the media containing 2 mM calcium. The findings showed that glycoside-4 could decrease Ca^2+^ influx in Etx-treated MDCK cells, as FACS analysis depicted a drastic reduction of Fluo-4 AM intensity, which was similar to the untreated control (Fig. 2F). Further, fluorescence microscopy analysis also validated the decrease in Fluo-4 AM intensity in the glycoside-4-treated MDCK cells compared to Etx (Fig. 2G). Together, our results authenticate that glycoside-4 hinders the process pore formation (Fig. 2B), thus blocking Ca^2+^ ingress.

### Glycoside-4 reverses the cell death caused by Etx and restores normal cellular homeostasis

**Fig. 3.**
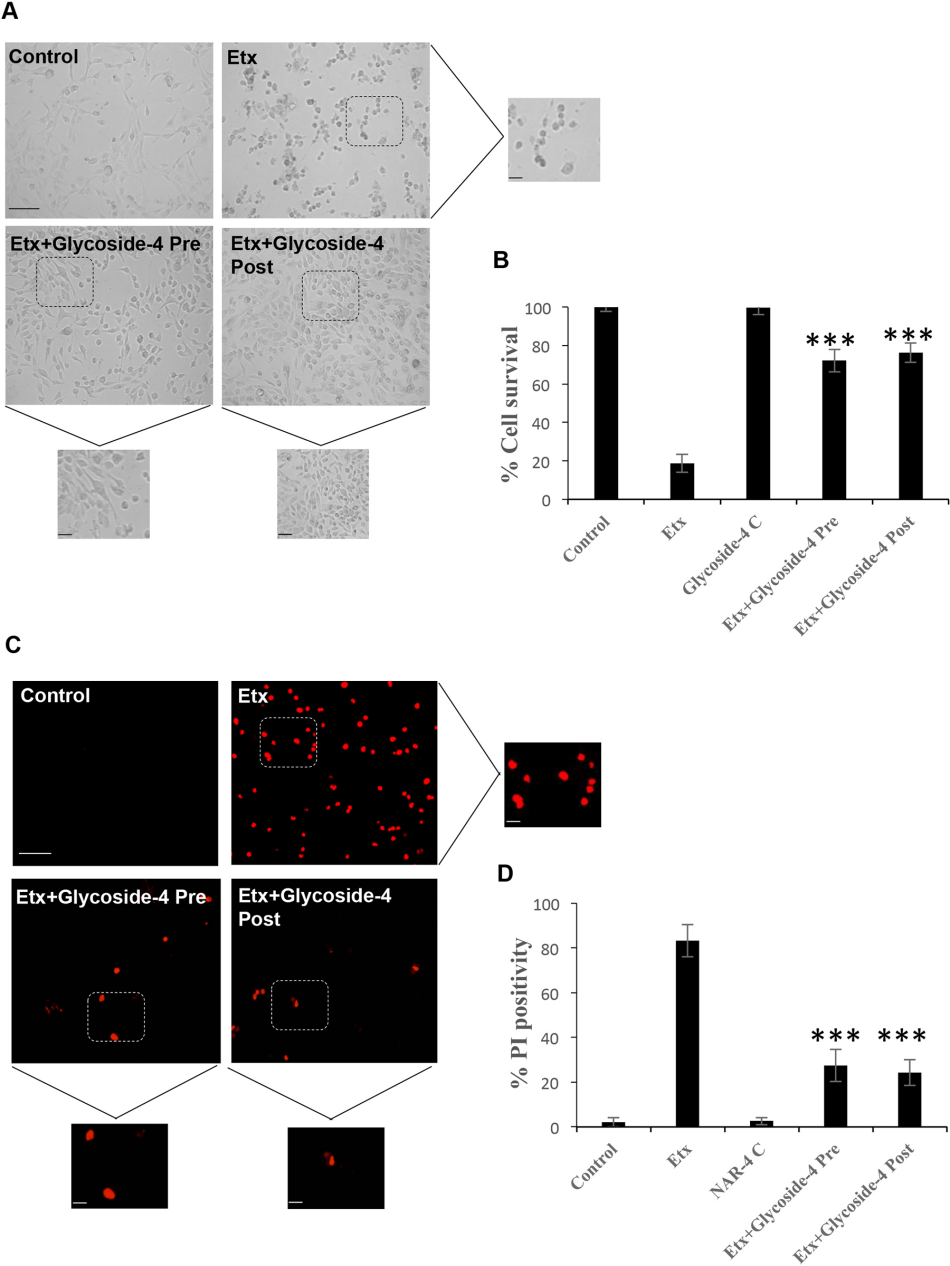
***In vitro* analysis of glycoside-4 reveals an enhanced level of protection in Etx-treated MDCK cells.** (A) Morphometric analysis was carried out to determine the ability of glycoside-4 to decrease the toxicity of MDCK cells in the presence of Etx at pre- or post-treatment conditions. Glycoside-4-treated cells showed intact surface architecture compared to Etx-treated cells. Enlarged areas of the corresponding images are shown. Scale bar: 20 μm. (B) Quantitative analysis by MTT shows rescue of MDCK cells treated with glycoside-4 in comparison to the Etx-only cells. ***P<0.005, one-way ANOVA followed by post-hoc (Bonferroni) test was performed. (C) The ability of glycoside-4 to decrease the Etx-induced PI influx in MDCK cells was detected. Glycoside-4-treated cells showed a drastic decrease in PI uptake compared to Etx-treated cells. Scale bar: 20 μm. Enlarged areas of the corresponding images are shown. (D) PI count shows a sharp decrease in positivity compared to the Etx-treated cells. ***P<0.005, one-way ANOVA followed by post-hoc (Bonferroni) test was performed.

Existing evidence suggests that calcium influx can be associated with both apoptosis (Ditsworth et al., 2007; Kennedy et al., 2009) and necrosis (Scaffidi et al., 2002). Since glycoside-4 could block the Ca^2+^ ingress in the presence of Etx, we hypothesize that it might also normalize the subcellular phenotypes and restore healthy cellular homeostasis. To ascertain the reversal of cell death, we performed morphometric analysis using light microscopy and propidium iodide (PI) staining in both pre-treatment (incubation of Etx with glycoside-4) and post-treatment (without incubation of Etx and glycoside-4) conditions. The results show an intact cellular architecture (Fig. 3A) and increase in cell survival in glycoside-4-treated cells upon Etx challenge (Fig. 3B). Staining for PI also showed higher levels of cellular protection by glycoside-4, as evidenced by a drastic reduction in PI^+^ cells (Fig. 3C,D), with no major difference between pre- and post-treatment with glycoside-4, indicating cellular protection.

**Fig. 4.**
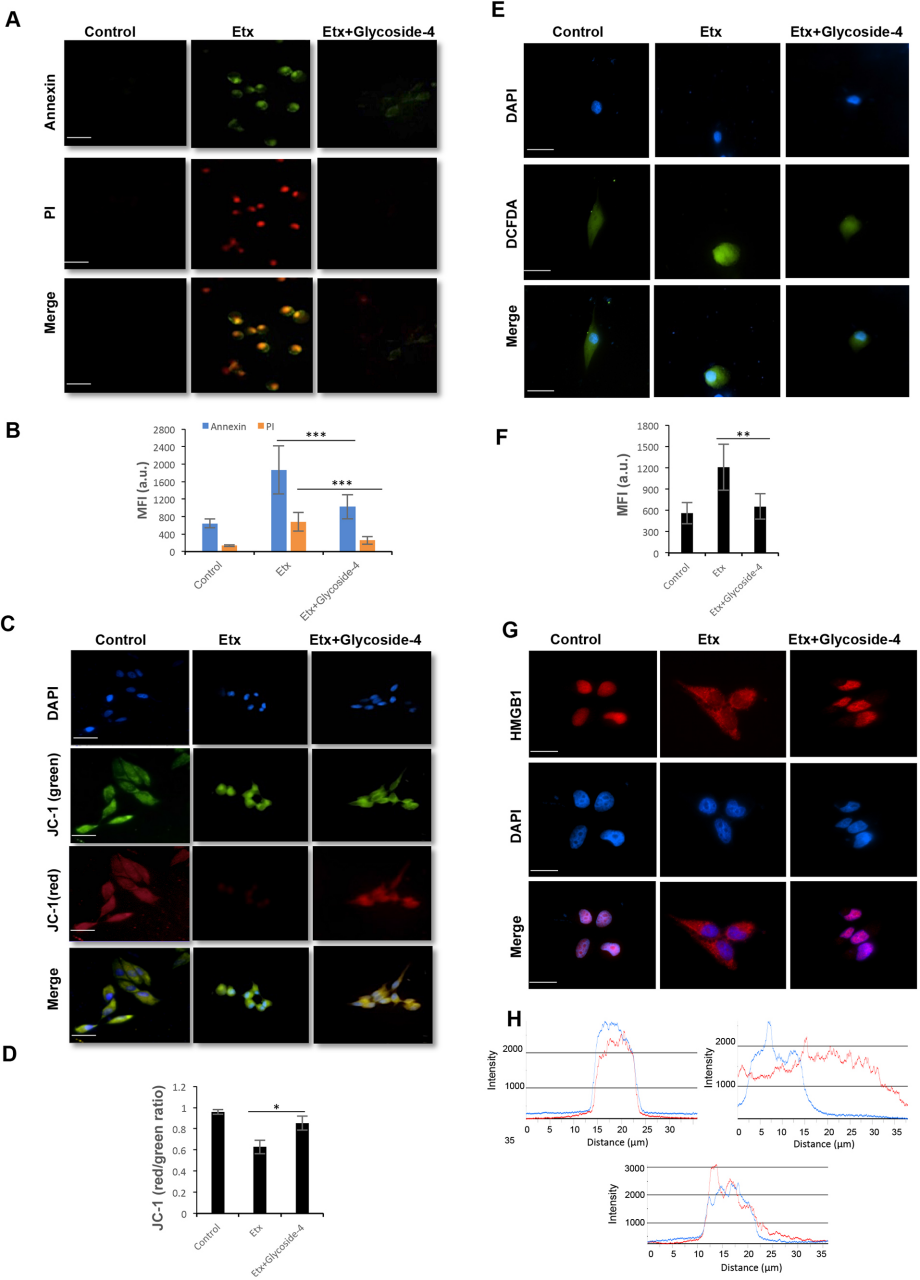
**Glycoside-4 protects MDCK cells from Etx-triggered cell death.** (A) MDCK cells were treated with Etx or in combination with glycoside-4, and the annexin V staining of exposed phosphatidyl serine on the outer surface and PI entry were observed. The toxin-treated cells showed annexin and PI (annexin^+^/PI^+^) dual positivity, whereas glycoside-4-treated cells showed the absence of both the markers (annexin^−^/PI^−^). Scale bars: 20 μm. (B) Bar graph depicts mean fluorescence intensity (MFI) for annexin and PI. Statistical significance (***P<0.005, Student's *t*-test) between indicated groups is shown. Error bars represent mean±s.d. (C) MDCK cells were loaded with JC-1 and the effect on mitochondrial membrane depolarization (ΨΔm) was observed. Representative images show accumulation of monomeric JC-1 (green) in response to Etx, indicating disruption of ΨΔm. But, upon glycoside-4 treatment, the potential was restored as represented by JC-1 aggregates (red), comparable to control cells. Scale bars: 20 μm. (D) Bar graph depicts the red (aggregates)/green (monomers) ratio. Statistical significance is shown between indicated groups (*P<0.05, Student's *t*-test). Error bars represent mean±s.d. (E) MDCK cells were treated with DCFDA and the accumulation of ROS was observed. Images show increased production of ROS in the Etx-treated cells, whereas glycoside-4-treated cells displayed lower intensity compared to Etx. Scale bars: 20 μm. (F) Bar graph represents MFI for DCFDA. Statistical significance (**P<0.01, Student's *t*-test) between indicated groups is shown. Error bars represent mean±s.d. (G) The Etx-treated cells showed a drastic translocation of HMGB1 from the nucleus to the cytoplasm, indicating necrotic death. Upon glycoside-4 treatment, cells show localization of HMGB1 largely within the nucleus. Scale bars: 20 μm. (H) Colocalization graphs of DAPI (blue) and HMGB1 (red) are shown.

Previous studies have shown that Etx can induce apoptosis, manifested by exposure of phosphatidylserine (PS) residues on the outer cell surface (Fadok et al., 1998). Further, to validate whether glycoside-4 treatment has any impact on flipping of PS in the presence of Etx, we stained Etx-treated MDCK cells with annexin V dye (which binds to exposed PS residues) and PI. Etx exposure led to flipping of PS residues, leading to cell permeabilization and death, as detected by enhanced annexin V^+^/PI^+^ cells, a typical feature of late apoptotic/necrotic stages (Fig. 4A). Upon treatment with glycoside-4, cells showed no exposure of PS on the outer membrane, as indicated by the absence of annexin V^+^/PI^+^ cells (Fig. 4A), nullifying the Etx-triggered late apoptotic/necrotic responses. Fluorescence intensity analysis revealed a substantial decrease for annexin V and PI in glycoside-4-treated cells compared to Etx (Fig. 4B).

Another feature of necrosis is the disruption of ΨΔm, which is often correlated with reactive oxygen species (ROS) production and subsequent cell death (Zong and Thompson, 2006). Hence, to evaluate the effect of Etx on mitochondrial disruption and ROS generation, we used the ΨΔm indicator dye JC-1 and ROS indicator dye 2′,7′-dichlorofluorescin diacetate (DCFDA). Upon treatment with Etx, the cells showed a rapid decrease in ΨΔm in cells, as was evident by an increase in green intensity (monomers) as compared to control (Fig. 4C), whereas glycoside-4 could stabilize ΨΔm in Etx-treated MDCK cells, resulting in accumulation of oligomeric JC-1 complexes (Fig. 4C). A clear shift in the ratio of red (oligomer)/green (monomer) could be seen in the Etx-treated MDCK cells, whereas an intact potential comparable to the control could be observed in the glycoside-4-treated MDCK cells (Fig. 4D).

Similarly, glycoside-4-treated cells exhibited decreased levels of ROS, as shown by diminished green staining compared to Etx treatment (Fig. 4E,F). ROS generation is often correlated with PARP activation, leading to the release of high mobility group box 1 (HMGB1), a protein that is usually present in the nucleus during apoptosis but translocates to the cytoplasm during necrosis (Ditsworth et al., 2007; Scaffidi et al., 2002). To further understand whether glycoside-4 could antagonize the HMGB1 translocation in the presence of Etx, we performed fluorescence microscopy. Upon glycoside-4 addition, HMGB1 largely remained in the nucleus with no detectable shift to the cytosol, indicating the absence of cellular necrosis (Fig. 4G). The intensity plot indicated the colocalization of DAPI (blue) and HMGB1 (red) in both the control and with glycoside-4 treatment (Fig. 4H), whereas no colocalization of DAPI and HMGB1 could be observed in cells treated with Etx alone (Fig. 4H).

Taken together, our data are in line with the previous studies (Chassin et al., 2007; Popoff, 2011) that Etx causes cell death of MDCK cells accompanied by membrane blebbing and increased Ca^2+^ levels, followed by mitochondrial depolarization, ROS generation and HMGB1 release from the nucleus to cytoplasm. In contrast, glycoside-4 rescues Etx-treated MDCK cells by inhibiting all the characteristic features of cell death.

### Glycoside-4 protects the Etx-sensitized C57BL/6 mice from Etx lethality

**Fig. 5.**
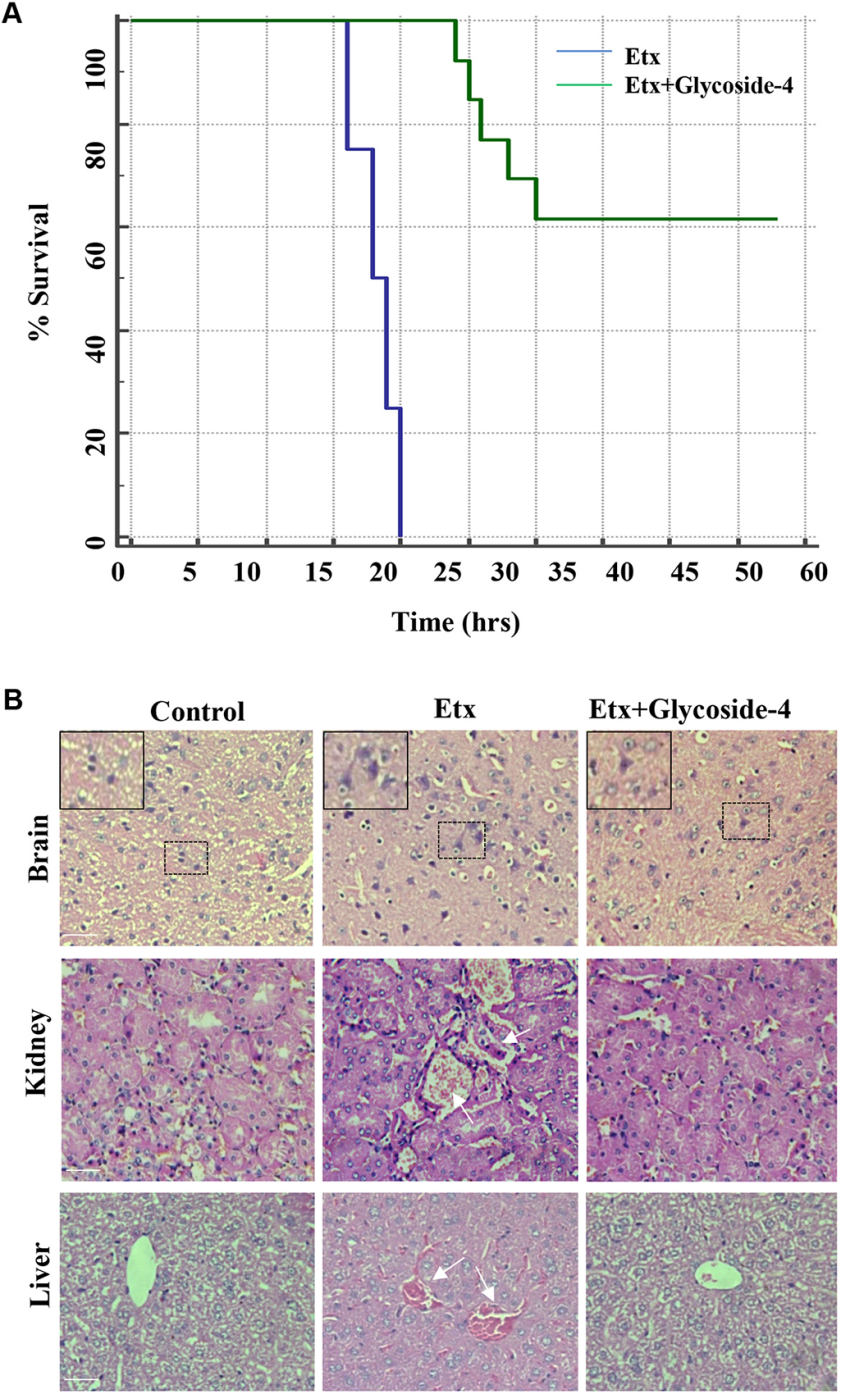
**Glycoside-4 protects C57BL/6 mice from Etx lethality.** (A) Two groups of mice were treated with either Etx (*n*=15) or in combination with glycoside-4 (*n*=15) and survival was determined until 48 h. Kaplan–Meier curves showing significant difference in survival between Etx and Etx+glycoside-4 groups. *P*<0.0001, log-rank test. (B) H&E staining was carried out to detect the histopathological alterations in the brain, kidney and liver of mice. The PBS group had healthy architecture in all cases, whereas the Etx group showed severe abnormalities in all the organs. However, the glycoside-4-treated group showed improved tissue structures, which were comparable to the PBS group. Boxed areas are enlarged as indicated in the top left corner of each image in brain. Healthy neuronal structure (PBS group), degenerated axonal structures (Etx group) and improved neuronal architecture (glycoside-4+Etx group), similar to the PBS group, could be seen. White arrows represent necrosis in glomerulus and tubules (kidney, Etx group) and blood sinusoids in liver (liver, Etx group). Scale bars: 20 µm.

To evaluate the effect of glycoside-4 in Etx-challenged C57BL/6 mice, we injected 150 ng/kg body weight, a median lethal dose (LD_50_), of purified Etx intraperitoneally (i.p.). Initially, to evaluate the toxicity of glycoside-4 in mice (*n*=5), doses ranging from 50 to 250 mg/kg body weight were administered (i.p.) and monitored for 48 h. The control group received only PBS (*n*=5). No mortality or clinical signs (seizures, convulsions and depression) were detected in both the groups during this period. The protective efficacy of the compound was determined by treating the mice with glycoside-4 (50 mg/kg body weight) and 4×LD_50_ dose of the toxin (*n*=15). The Etx mice received the same toxin dose (4×LD_50_, *n*=15) and the animals were observed until 48 h post-injection. All the Etx mice died between 15 and 20 h (*n*=15/15, 100%), whereas a 40% (*n*=6/15) death rate was seen in mice treated with Etx and glycoside-4 (Fig. 5A). This surviving group showed symptoms such as isolation, circling with dizziness and lethargic behaviour for 10-12 h before full recovery. No mortality was observed beyond this point. The Etx-treated mice were dissected after death and the surviving mice in the glycoside-4-treated group were sacrificed by cervical dislocation according to the approved institutional animal ethics procedure. Brain, liver and kidney were isolated and subjected to histopathological analysis. Haematoxylin and eosin (H&E) staining of the mouse organs revealed gross pathophysiological changes in the Etx group (*n*=15/15), which mainly affected the kidney, causing enterotoxaemia, followed by brain and liver damage. The changes in the brain included necrosis of neurons, focal gliosis, axonal degeneration (arrows, Fig. 5B) and inter-cytoplasmic vacuoles (Fig. 5B). Glomerular disruption, thickening of basement membrane and necrosis of the distal convoluted tube (DCT) were evident in kidney (Fig. 5B). Central vein congestion, blood sinusoids, and binuclear and giant cells were distinct in the liver (Fig. 5B). By contrast, Etx-challenged mice that had glycoside-4 treatment demonstrated protected axonal structures without any instance of focal gliosis and inter-cytoplasmic vacuoles (Fig. 5B) (*n*=9/15, 60%). Swelling of brain was detected in Etx-treated mice, whereas the glycoside-4-treated mice showed intact brain structure, comparable to healthy brains. Similar improvements were observed in glycoside-4-treated liver (*n*=9/15, 60%). Only minimal changes, such as a slight increase in glomerular volume and narrowing of the Bowmen's capsule, were seen in glycogen-4 treated kidneys (Fig. 5B) (*n*=12/15, 80%), with the entire tissue structure remaining largely intact. These findings indicate that glycoside-4 can protect multiple affected organs of C57BL/6 mice from Etx-induced lethality.

## DISCUSSION

According to the CDC list of potent bio-weapons, *C. perfringens* Etx has been categorized as a class B type of lethal neurotoxin that poses potential threat to domesticized animals and humans (CDC, 2000). Numerous therapeutic approaches have been undertaken in the past, including: development of neutralizing antibodies (McClain and Cover, 2007), generation of dominant-negative mutants of Etx (I51C, A114C, V56C and F118C) (Pelish and McClain, 2009) and the discovery of small molecules through library screening (Lewis et al., 2010). None of these approaches has been successful and there are no human interventions available to date against the potential bio-terrorizing effects of Etx.

Structural insights have revealed three crucial domains in Etx: (1) membrane binding, (2) oligomerization and pore formation, and (3) monomer-monomer interaction (Knapp et al., 2009). Previous studies have reported three small-molecule inhibitors against Etx, namely, N-cycloalkylbenzamide, furo[2,3-b]quinoline and 6H-anthra[1,9-cd]isoxazol, from a library of 1,51,616 compounds (Lewis et al., 2010). However, none of them could inhibit Etx binding or oligomerization. Emerging studies have shown that sugar-based derivatives can inhibit the production of *C. perfringens* alpha-toxin (PLC) and perfringolysin O (PFO) toxin, responsible for gas gangrene (Ferreira et al., 2016; Méndez et al., 2012). Other glucose analogues, such as 2 deoxy-D-glucose and alpha-methyl-glucoside, can also inhibit PLC production (Duncan and Cho, 1972). Another study shows that monovalent Shiga toxin (Stx) ligands of phosphatidylethanolamine dipalmitoyl-Gb_3_ (Gb_3_-PEDP) and galabiosyl (Gb_2_)-PEDP neutralize Stx cytotoxicity. This neutralization mechanism involves formation of liposomes and clustering of sugar units (Neri et al., 2007). Taking inspiration from these studies, we have designed and synthesized a library of glycoside derivatives (1-12) (Fig. S1A,B and Table S1). It is noteworthy that the β-barrel structure of domain II plays a major role in Etx-mediated pore formation; thus, we hypothesized that targeting domain II might lead to effective blocking of Etx activity.

To determine the preferential ligandability of domain II against all the glycosides, we have generated a heptameric pre-pore structure of Etx based on the crystal structure of aerolysin and validated its stability by MD simulation (Fig. 1A,B and Fig. S2A). During the revision of this paper, the Etx pore structure was published (Savva et al., 2019). In that study, the authors have proposed a hypothetical pre-pore to study the structural rearrangements required for transition to pore state. However, since the C-terminal peptide (CTP) was not removed during the pre-pore construction, the authors concluded that the CTP severely obstructs oligomerization. However, in our pre-pore, the N- and C-terminal residues (which are cleaved by trypsin during activation) were removed during the construction of the structure. Since removal of the CTP is required for oligomerization, docking with our pre-pore structure would closely resemble the actual pre-pore to pore scenario. Hence, we *in silico* docked all the glycosides with our heptameric pre-pore, which ascertained a druggable pocket in the β-hairpin region, as most of the compounds showed preferential binding to the same (Fig. S2C and Table 1). Out of 12 molecules, three of them (glycoside 4, 5 and 12) showed very similar binding energies (−6.9, −7.0 and −6.9 kCal/mol, respectively). Although all these molecules showed no toxicity (Fig. S2D,E), screening for anti-Etx activity revealed glycoside-4 as the lead molecule (Fig. S3A and Fig. 1E). It is assumed that the differential ability of glycoside-4 might be due to its side-chain-based structural composition. Notably, derivatives of monosaccharides and disaccharides can get self-assembled, because of poly-hydroxy groups, and are able to form aggregates or micelle-like structures in aqueous medium due to possible hydrophobic interactions by the carbon chain (Sonoda and Skaf, 2007; Vidyasagar et al., 2011). Glycoside-4 has a butyl group with a linear side chain, which suggests a robust possibility of forming stable micelles, while glycoside-5 has a branched carbon chain with a strong possibility of reduced hydrophobic interactions, and thus might form fewer stable micelles. However, glycoside-12 has an -OH group replaced by NHAc along with a longer carbon chain, suggesting an imbalanced hydrophobic-hydrophilic conformation. This assumption on glycoside-4 structure was further validated by evaluation of its stable self-aggregation (or micellization) attribute using fluorescent spectroscopic analysis. Glycoside-4 forms micelles with a CMC of 15.625 µM (Fig. 2C,D) in water. It is evident that the pocket residues of the β-hairpin region in domain II of Etx, such as Thr138, Cys139, Glu191, Thr193, Asn195 and Val196, could make ten strong intermolecular H-bonds with glycoside-4 (Fig. 1C and Fig. S3B). Thus, micelle of glycoside-4 might enforce strong steric hindrance on the heptameric form of Etx following binding to the residues in the β-hairpin, leading to abrogated oligomerization. Further, analysis of oligomerization *in vitro* corroborated this *in silico* docking analysis, as the formation of heptamers was substantially impaired in Etx-challenged MDCK cells in the presence of glycoside-4, as evidenced by reduced intensity of the heptameric complex in immune blot (Fig. 2A). To further authenticate the impact of glycoside-4 on biophysical interactions of Etx monomer-monomer assembly, we performed SPR. The findings revealed strong obstruction in monomer-monomer interaction in the presence of glycoside-4, with no effect on Etx-monomer alone (Fig. 2E). Precisely, this finding strongly coincided with our findings, which highlighted that glycoside-4 hindered stable/functional oligomer formation (Fig. 2A). However, *in silico* docking of glycoside-4 with Etx monomer revealed weaker binding, which could not be effectively detected in SPR.

Next, to evaluate whether blocking of oligomerization by glycoside-4 can also abolish pore formation, we added Etx treatment with glycoside-4 to liposomes and analyzed pore formation using TEM (Fig. 2B). Interestingly, liposomes treated with Etx alone displayed huge ‘pizza-slice shaped tearings’, whereas, upon glycoside-4 treatment, Etx could not make oligomers/pores in the majority of cells, as visualized in TEM (Fig. 2B). These data suggested that glycoside-4 might block the pore formation by binding to the residues in the β-hairpin loop, in turn sterically hindering the insertion loops required for pore formation (Fig. 1C and Fig. S3B).

Since glycoside-4 could hamper oligomerization, we hypothesized that glycoside-4 could restore the cellular homeostasis modulating the downstream cellular and molecular responses. To prove this hypothesis, we have established an Etx-treated MDCK model to study several features of cell death. Previous studies have indicated that Ca^2+^ influx is often correlated with the induction of apoptosis (Mattson et al., 2003) and also involved in programmed necrosis as evident in the case of PLC (Kennedy et al., 2009). In the case of β-PFTs such as Etx, binding and oligomerization results in an increase of Cl^−^, Na^+^ and Ca^2+^ with a decrease in intracellular K^+^ (Petit et al., 1997). The Etx-mediated MDCK cell death model could demonstrate similar phenotypes, as it triggered a sequential process of cell death via increased Ca^2+^ influx ultimately leading to PI positivity (Fig. S4D). Along with this, the model also displayed other features of cell death, including membrane blebbing (Fig. S4C), increased annexin V^+^/PI^+^ staining, destabilization of ΨΔm and translocation of HMGB1 from the nucleus to the cytoplasm. Interestingly, upon treatment with glycoside-4, all these features of cell death got reversed, leading to healthy cellular homeostasis (Fig. 4). This included a substantial reduction in Ca^2+^ influx as shown by reduced Fluo-4 AM intensity (Fig. 2F,G), indicating a blockage of pore formation, stabilization of ΨΔm (Fig. 4C,D), impairment of ROS generation (Fig. 4E,F) and finally abrogation of HMGB1 translocation (Fig. 4G,H). Recently, a flavanone, 5,8-dimethoxy-6-methyl-7-hydroxy-3-3(2-hydroxy-4-methoxybenzyl) chroman-4-one (58-F), extracted from *Ophiopogon japonicas* has been reported to play a similar role (Yan et al., 2016). This flavonoid protects the H_2_O_2_-induced damage in hepatocytes by decreasing the calcium concentration and suppressing the PLCγ1-IP_3_R-SOC pathway, leading to reduced HMGB1 release. Since HMGB1 is a signature for necrosis and its release to the cytosol is implicated in cell death (Lu et al., 2014; Scaffidi et al., 2002), abrogation of its translocation in Etx-challenged MDCK cells following glycoside-4 treatment suggests a remarkable restoration of healthy cellular homeostasis (Fig. 4G,H).

Based on all these observations, we then tested the ability of glycoside-4 to rescue the Etx-challenged C57BL/6 mice. Initially, the lethality of Etx was very rapid owing to the intravenous (i.v.) exposure (within 30 min), which was approximately ten times higher than in mice exposed to Etx at the same concentration via i.p. The LD_50_ of the toxin preparation was found to be 150 ng/kg body weight, which closely resembles the previously reported LD_50_ (Sakurai and Kobayashi, 1995). This determination of LD_50_ is essential as the difference in purification protocols can lead to the variations in the LD_50_ concentrations (Minami et al., 1997). Treatment with different doses of glycoside-4 was optimized and 1 mg/mouse dose provided the best protection. This dose could protect 60% (*n*=9/15) of the mice challenged with 4×LD_50_ of Etx with a single dosage (Fig. 5A). Moreover, glycoside-4 could also protect vital organs such as the brain, liver and kidney from Etx lethality (Fig. 5B). To our knowledge, this is the first report of a glycoside derivative that can bind to a pocket in domain II and block oligomerization and membrane insertion, thus providing both *in vitro* and *in vivo* protection against Etx. In summary, due to non-toxicity and rapid anti-Etx activity, we propose that glycoside-4 could be developed into a first line of defence against Etx lethality and a strong antidote against the bio-terrorizing effects of Etx.

## MATERIALS AND METHODS

### Glycoside synthesis general procedure

Amberlite IR 120-H^+^ resin (400 mg) was added to a pre-stirred solution of glucose 1 (200 mg, 1.11 mmol) in neat alcohol (5-10 ml). The resulting mixture was stirred at 100°C for 24 h. After completion of the reaction, reaction mixture was cooled down to room temperature, and filtered to remove the resin. The filtrate was evaporated under reduced pressure to obtain compound glycoside-1 to glycoside-6 as white solid in an acceptable to good yield. Amberlite IR 120-H^+^ resin (400 mg) was added to a pre-stirred solution of N-acetyl glucosamine-2 (200 mg, 0.9 mmol) in neat alcohol (5-10 ml). The resulting mixture was stirred at 100°C for 24 h. After completion of the reaction, reaction mixture was cooled down to room temperature, and filtered to remove the resin. The filtrate was evaporated under reduced pressure to obtain compound glycoside-7 to glycoside-12 as white solid in an acceptable to good yield. ^1^H NMR and ^13^C NMR spectra in CD_3_OD and D_2_O were recorded on a Bruker 400 MHz spectrometer at ambient temperature (^1^H recorded by 400 MHz and ^13^C recorded by 100 MHz). Proton chemical shifts are given in ppm relative to the internal standard (tetramethylsilane) or referenced relative to the solvent residual peaks (CD_3_OD: δ 3.31). Multiplicity was denoted as follows: s (singlet); d (doublet); t (triplet); q (quartet); m (multiplet); dd (doublet of doublet); dt (doublet of triplet); td (triplet of doublet); ddd (doublet of doublet of doublet), etc. Coupling constants (*J*) were reported in Hz. Column chromatography was performed by using silicagel 100-200 and 230-400 mesh. High resolution mass spectrometry (HRMS) spectra were determined from a quadrapole/Q-TOF mass spectrometer with an ESI source (supplementary text).

### Etx purification

The purification of Etx was carried out as described elsewhere (Goswami et al., 1996). In general, the *E. coli* M15 cells harbouring the pQE60 Etx were induced with 1 mM isopropyl-β-D-thiogalactoside (IPTG) for 5-6 h at 37°C. The soluble fraction containing Etx was purified using (DEAE)-Sepharose anion exchange chromatography (Amersham Pharmacia, NJ, USA). The proto-toxin was further activated to mature toxin (active form) by treatment with trypsin (Hunter et al., 1992). The protein concentration of mature toxin was measured using a BCA estimation kit (Pierce, Rockford, IL, USA) and used for all the experiments.

### Heptamer 3D construction

The Swiss PDB viewer software was utilized for construction of the heptameric unit. Using aerolysin toxin structure as a template (PDB: 5JZT), the monomer units were aligned at similar angles, which were rendered stable after energy minimization. We also used Super-align and TM-align commands on Pymol to structurally align the heptamer of 5JZT, the Cryo-EM structure of Aerolysin pore. 5JZT was aligned to the seven-monomer collection of 1UYJ, thus translating the monomers at similar angles to obtain a heptameric unit. Each heptameric unit of the protein was correctly labelled as chains A-G using in-house scripts. The amino acids were numbered according to UniProt (Q57398_CLOPF). The heptameric molecule was energy-minimized in MOE using CHARMM force field. The lead compound, glycoside-4, was used for further studies. All 12 compounds were drawn through the Sketch module of Molecular Operating Environment (MOE) (Montreal, Canada).

### MD simulations

MD simulations of both the energy-minimized monomer and heptameric models of Etx were performed in order to analyze the stability of the modelled oligomeric structure. We used GROMACS version 5.06, which is a versatile collection of programs and libraries for simulating molecular dynamics of large proteins and subsequently analyzing trajectory data. The MD simulation was carried out on a parallel processing internal server (Magus), which is a 60-node, 992-core IBM high performance cluster (HPC). The GROMOS 96 53a6 force field including all hydrogens, along with a simple point-charge (SPC) water model, was used for energy minimization of both monomeric and oligomeric models. The pre-equilibrated SPC water was added to a dodecahedral box, and the individual protein was then placed in the centre of the box. In total, 4,677,864 solvent molecules were externally added into the box in order to solvate the system. Furthermore, an optimal number of ions (Na^+^ and Cl^−^) was added by -neutral option to neutralize the whole protein. The protein and non-protein groups were energy minimized with a tolerance of 1000 kJ mol^−1^ nm^−1^ using the steepest descent method for 50,000 steps. All the bonds were constrained using the LINCS algorithm, and the simulation was performed under isothermal-isobaric ensemble (NPT) and canonical ensemble (NVT) conditions, using the v-rescale coupling algorithm and the Parrinello-Rahman coupling algorithm, which stabilized the temperature and pressure (*P*=1 bar, τ*P*=0.1 ps; *T*=300 K, τ*T*=0.1 ps). The smooth particles mesh Ewald (PME) method was used with a cut-off of 1.4 nm for electrostatic and van der Waals (vdW) interactions. The electrostatic interactions were calculated with PME using a grid spacing of 0.12 nm. Periodic boundary conditions (PBCs) were employed to eliminate surface effects. The final MD simulations were carried out with a time step of 2 fs and without any position restraints; 17,500,000 steps were performed for a total of 40 ns. The structural deviations were analyzed by computing RMSD and RMSF. The RMSD and RMSF graphs were plotted using XMgrace software (Weizmann, Rehovot, Israel).

### Docking studies

In accordance with a hydrophobic pocket within the monomeric structure of Etx (PDB: 1UYJ), a virtual grid was constructed for docking the compounds using the Autogrid module of AutoDockTools. This 3D grid was of 26×28×30 Å measured volume with default spacing and exhaustiveness level of 0.375 Å and 8, respectively. The exhaustiveness level was kept high to increase the time linearly and decrease the probability of not finding the minimum exponentially. The top-ranked poses of the protein with the compound were selected based on the lowest free binding energies. In accordance with proposed active sites lying in domain II of Etx, a virtual grid/space was constructed covering at least two monomeric units for docking the compounds using the Autogrid module of AutoDockTools. This 3D grid was of 40×38×36 Å measured volume with default spacing and exhaustiveness level of 0.375 Å and 8, respectively. We performed molecular docking studies of glycosides 1-12 via Autodock Vina to rationalize its activity against Etx for the monomer as well as heptamer. For all molecular visualization and H-bond analysis of the proteins/ligands and docked protein-ligand complex, PYMOL 1.7.4 software was used.

### MDCK cell culture

The MDCK cell line was maintained in DMEM (Gibco) supplemented with 10% FBS (Gibco) and 1% penicillin/streptomycin (Gibco).

### Cell viability

MDCK cells were cultured in 96-well plates at a density of 0.2×10^6^ cells/well to analyze the cytotoxic effects of purified Etx. Serial dilutions of the Etx mixed in DMEM containing 10% FBS (Gibco) and 1% antibiotic (Gibco) were incubated with MDCK cells in triplicates for 1 h at 37°C. The control wells had an equal volume of media. Cell viability was measured using MTT [3-(4,5-dimethylthiazol-2-yl), 2,5 diphenyltetrazolium bromide] (Himedia) and the absorbance was read at A_550_ using Bio-Rad iMark microplate reader (Bio-Rad)_._ Untreated cells were taken as 100% cell survival, and the viability for Etx-treated cells were calculated accordingly. In the case of pre-treatment, glycoside-4 and Etx were incubated for 30 min before addition onto the cells, whereas, in the case of the post-treatment condition, glycoside-4 and Etx were added to the cells simultaneously.

### Electrical cell impedance sensing

MDCK cells were grown to confluence in the 8W10E (Applied Biophysics) well arrays with each well having an area of 0.8 cm^2^ and 250 µm active gold-plated electrodes. Once the resistance reached a plateau, the cells were washed twice with DMEM containing antibiotics and subsequently the active Etx was added. The data was collected at 4000 Hz using ECIS Zθ (Applied Biophysics) for 24 h post-treatment and analyzed using the Applied Biophysics (abp) software.

### Live-cell video microscopy

MDCK cells with a density of 2×10^4^ grown on glass-bottom culture dishes (ibidi) were treated with Etx and the morphological changes were observed in real time using a Nikon confocal microscope (Nikon A1R). To access calcium influx and PI positivity, the cells were initially washed twice with PBS followed by addition of Fluo-4 AM (1:400, Thermo Fisher Scientific, F14201) and PI (1:1000, Thermo Fisher Scientific, V13242) to the media. The Etx was added, and the Ca^2+^ and PI changes were observed under a confocal microscope equipped with a temperature-controlled stage (Nikon A1R, Nikon). The time-lapse images were captured using a 1.4 numerical aperture lens and analyzed by Nikon ES elements software.

### Binding and oligomerization assays

To detect membrane binding, the cells were grown to confluence in a six-well plate and incubated with Etx and/or with glycoside-4 for 1 h at 4°C. The cells were washed once with PBS and scraped in the same buffer. The harvested cells were lysed by two cycles of freezing and thawing, followed by centrifugation at 8000 ***g*** for 5 min at 4°C. The crude pellet fraction was boiled along with SDS dye and continued with the western blot using anti-Etx (1:500) as the primary antibody followed by anti-mouse HRP (1:5000, Sigma Aldrich, A9044) as the secondary antibody. The blot was developed using Amersham ECL detection reagents (GE Healthcare). Similarly, to detect the formation of oligomers, the same protocol was used in which MDCK cells were incubated with Etx and/or with glycoside-4 for 1 h at 37°C instead of 4°C followed by western blotting. This protocol has been modified from Mathur et al. (2010).

### Preparation of liposomes

All the phospholipids and cholesterol were obtained from Sigma Aldrich. Liposomes were prepared by extrusion method using dipalmitoylphosphatidyl choline (DPPC) and cholesterol at 45:50 mol% as described (Shepard et al., 1998).

### Transmission electron microscopy

A total of 0.5 mg/ml lipid mixture containing DPPC and cholesterol at 50:50 mol% in buffer [20 mM HEPES (pH 7.4) and 40 mM NaCl] was treated with Etx alone or along with glycoside-4 for 30 min at room temperature. After incubation, the liposomes were transferred to coated electron microscope grids and negatively stained with 1% (w/v) urinyl acetate (Darst et al., 1988). Electron micrographs were recorded at different magnifications using a JEOL 2100F microscope (JEOL).

### Surface plasmon resonance

To determine the Etx interactions as well as glycoside-4 interaction with Etx, SPR was performed using Auto Lab Esprit SPR. Etx (7.8 μM) was immobilized on the surface of a nickel-charged NTA SPR chip. Interaction analysis was studied by injecting Etx, glycoside-4 and Etx along with glycoside-4 over the chip surface, with an association and dissociation time of 300 and 150 s, respectively. HEPES buffer was used both as immobilization and binding solutions. The surface of the sensor chip was then regenerated with 50 mM NaOH solution. Data were fit by using Auto Lab SPR Kinetic Evaluation software provided with the instrument.

### FACS assays

MDCK cells were loaded with Fluo-4 AM and, after an incubation period of 30 min in DMEM medium, cells were washed. Etx was added to MDCK cells, and the increase in fluorescence was analyzed by flow cytometry by FACS Calibur (Becton & Dickinson) using Cell Quest software. Calcium ionophore (Sigma) was used as a positive control. For assessment of extracellular calcium, the assay was performed in the presence and absence of 1 mM EGTA (Sigma). To analyze inhibition by anti-Etx antibody, Etx was incubated with anti-Etx antibody or pre-immune antibody, prior to addition to MDCK cells. Similarly, to check for the inhibition by glycoside-4, Etx and glycoside-4 were incubated prior to addition to MDCK cells and analyzed by FACS Calibur (Becton & Dickinson).

### Annexin-PI and mitochondrial membrane potential assays

MDCK cells were treated with Etx and/or glycoside-4 and incubated for 1 h at 37°C. The cells were washed with PBS and stained with annexin V-FITC (1:400, Thermo Fisher Scientific, V13242) and PI (1:1000, Thermo Fisher Scientific, V13242) and imaged. Similarly, staining for the ΨΔm indicator JC-1 (1:400, Thermo Fisher Scientific, M34152) was carried out after incubation with Etx in the presence or absence of glycoside-4 for 1 h using the manufacturer's protocol and imaged using FITC and TRITC filters under the Nikon Ti microscope (Nikon).

### DCFDA staining

A total of 2×10^4^ MDCK cells were grown on the glass-bottom culture dishes and incubated at 37°C overnight. The cells were treated with Etx or in combination with glycoside-4 for 1 h at 37°C. After incubation, the cells were washed and stained with DCFDA (2-7-dichlorodihydrofluorescein diacetate) (1:300, Thermo Fisher Scientific, I36007) followed by imaging at 485 nm excitation filter and 535 nm emission filter under the Nikon Ti microscope (Nikon).

### HMGB1 assays

MDCK cells were grown on the sterile coverslips (Borosil) and incubated for 12 h at 37°C. The cells were treated as described in the culture section, and the coverslips were fixed with 2.5% paraffin. HMGB1 (1:500, Thermo Fisher Scientific, PA1-16926) was used as primary antibody followed by addition of anti-rabbit Alexa Fluor 594 (1:500, Thermo Fisher Scientific, A21429) as the secondary antibody. The coverslips were mounted on a glass slide using DAPI antifade (Thermo Fisher Scientific, P36931) and imaged under the Nikon Ti microscope (Nikon).

### Animal experiments

BALB/c mice (4-5 weeks) were injected with the activated Etx (10 μg/mouse) and the anti-sera was collected. C57BL/6 mice weighing about 20-25 g (5-7 weeks) of both sexes were used for the Etx and glycoside study. All the animals were obtained from the animal house of the National Institute of Immunology (NII), Aruna Asaf Ali Marg, New Delhi, India. The animals had *ad libitum* access to food and water, and were kept under standard laboratory conditions (12 h/12 h light-dark cycle, temperature 22±2°C). The Animal Ethics Committee of the institute (NII) approved the animal usage and procedures (IAEC Code #288/11). The control mice received only PBS and all the dilutions were prepared in PBS. The route for injection was intraperitoneal (i.p.) in all the cases.

### Toxin challenge experiments

In order to determine the LD_50_ of the toxin preparation, we used serial dilutions of toxin ranging from 1 µg/mouse to 0.01 µg/mouse. A total of eight mice received 200 µl of toxin (i.p.) of each dilution in PBS. Negative control mice were treated with PBS only. All the animals were monitored for 72 h post-injection. For the challenge experiments, the Etx+glycoside-4 group (*n*=15) received a single dose of 2×LD_50_, 4×LD_50_ and 6×LD_50_ of the toxin (i.p.) along with the 50 mg/kg body weight drug. The Etx control and PBS control were kept in all the cases. All the animals were monitored for 48 h post-injection. The mice were sacrificed at the end of the duration by cervical dislocation, and the brain, kidney and liver were removed for H&E staining.

### H&E staining

The brain, kidney and liver from all the groups (PBS, Etx and Etx+glycoside-4) were removed rapidly and stored in freshly prepared 10% neutral buffered formalin (Sigma), processed and embedded in paraffin. The sections were 3 µm thick and cut on the sagittal plane. These were stained with H&E for histopathological alterations. All the images were taken using the Nikon light microscope and processed using the Nikon software.

### Statistical analysis

One-way analysis of variance (ANOVA) followed by Bonferroni test was used for comparison. When appropriate, Student's *t*-test was performed wherever applicable. *P*-values of <0.05, <0.01 and <0.005 were considered significant (denoted by *, ** and ***, respectively). Results represent mean±s.d. of at least three independent experiments.

## Supplementary Material

Supplementary information
