# Supplementary material for: A small bioactive glycoside inhibits epsilon toxin and prevents cell death
Source: Dis Model Mech. 2019 Oct 10;12(10):dmm040410. doi: 10.1242/dmm.040410 (PMC6826021; doi:10.1242/dmm.040410)
Supplement: Supplementary information [file dmm-12-040410-s1.pdf]

A

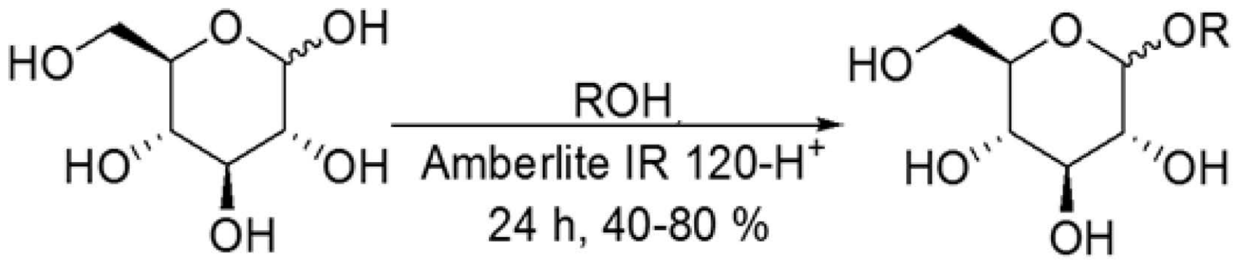

1

Glycoside-1 R=Ethyl, Glycoside-2 R=Propyl
Glycoside-3 R=Isopropyl, Glycoside-4 R=Butyl
Glycoside-5 R=Isoamyl, Glycoside-6 R=Hexyl

B

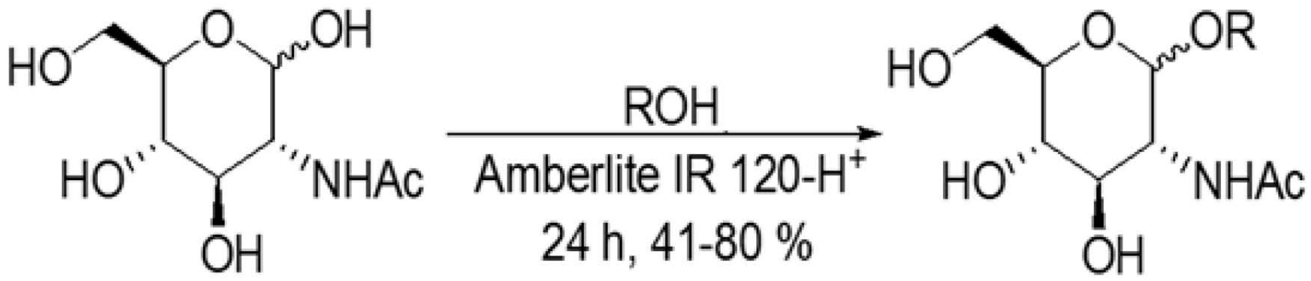

2

Glycoside-7 R=Ethyl, Glycoside-8 R=Propyl
Glycoside-9 R=Isopropyl, Glycoside-10 R=Butyl
Glycoside-11 R=Isoamyl, Glycoside-12 R=Hexyl

Fig S1: Scheme for synthesis of glycosides. A) Synthesis of designed glycosides was carried out with commercially available D-glucose 1 and D-glucosamine 2 coupling with various short to long chain alcohols under acidic condition. The scheme overview is represented. B) The alkyl glycoside of D-glucosamine Glycoside-7 to Glycoside-12 were prepared by refluxing D-glucosamine 2 with corresponding alkyl alcohols.

Disease Models & Mechanisms • Supplementary information

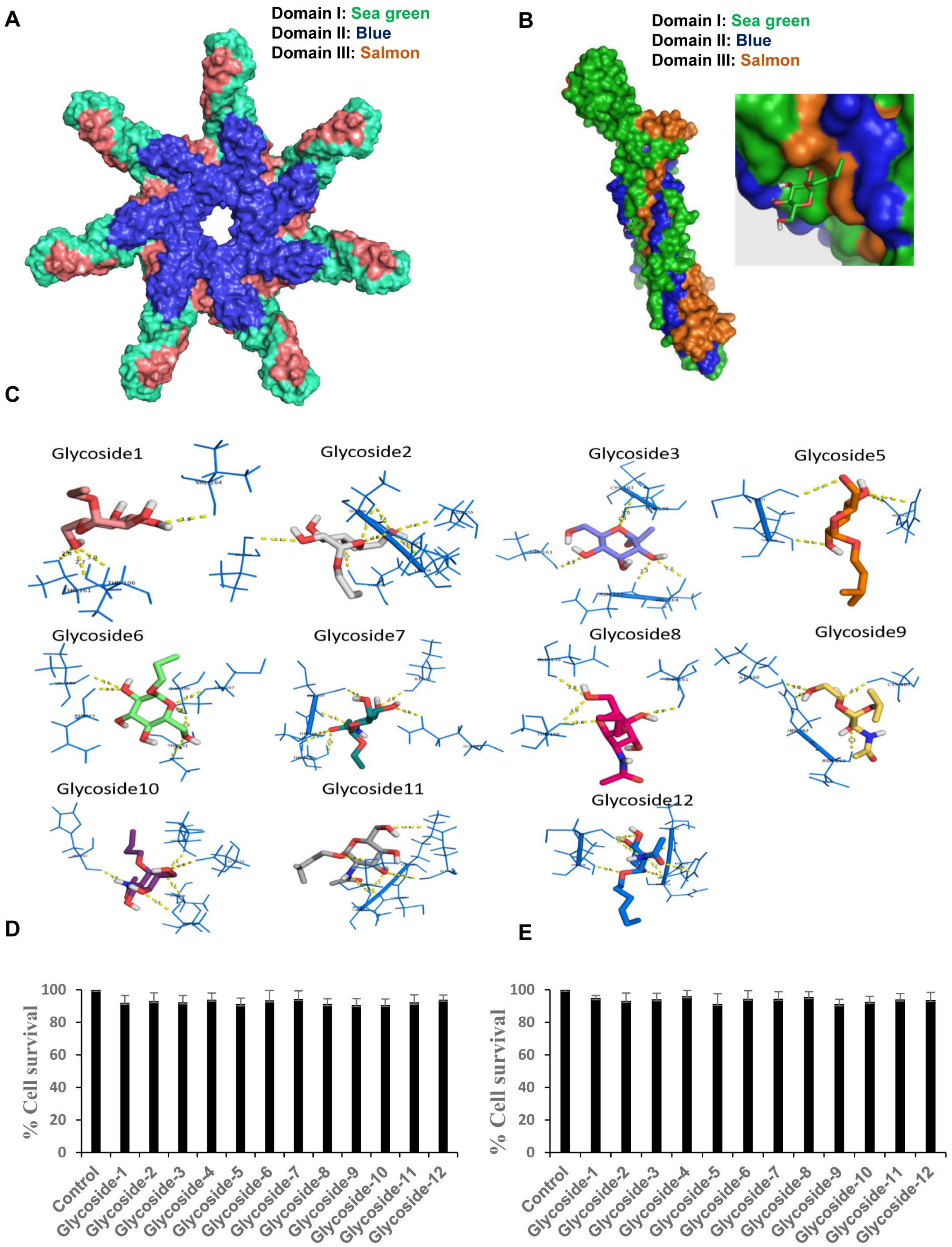

Disease Models & Mechanisms • Supplementary information

Fig S2: *In silico* generation of Etx heptamer and Glycoside-4 docking with Etx monomer and heptamer. A) Construction of Etx heptamer from the monomeric form (1UYJ) is represented. B) **Glycoside-4** binding to monomeric structure of Etx. C) The docking of the glycosides with the heptameric Etx is shown. D) All the synthesized glycosides (1-12) were screened for their cytotoxicity (100 µM) in HepG2 cells using MTT assay. E) All the synthesized glycosides (1-12) were screened for their cytotoxicity (100 µM) in MDCK cells using MTT assay.

A

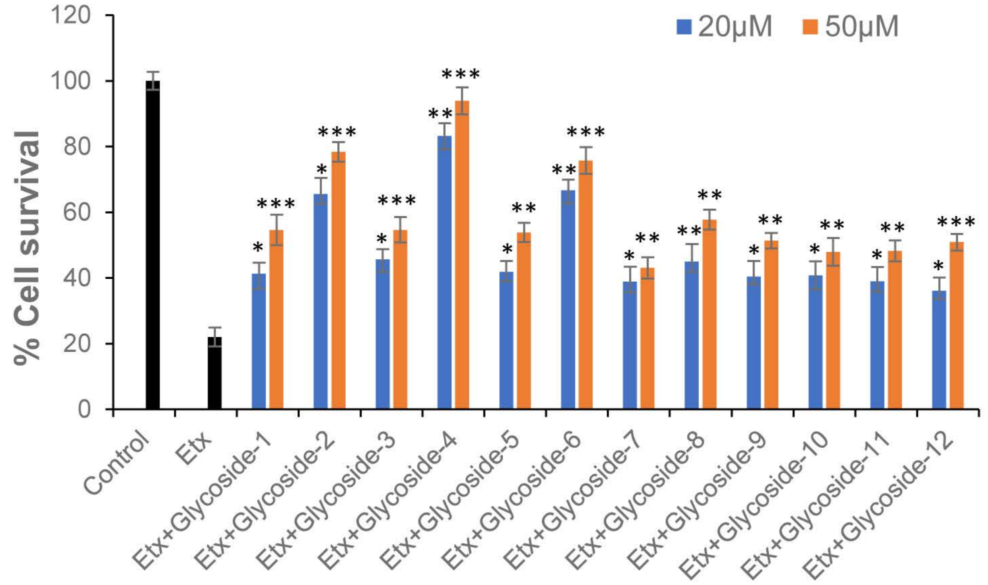

B

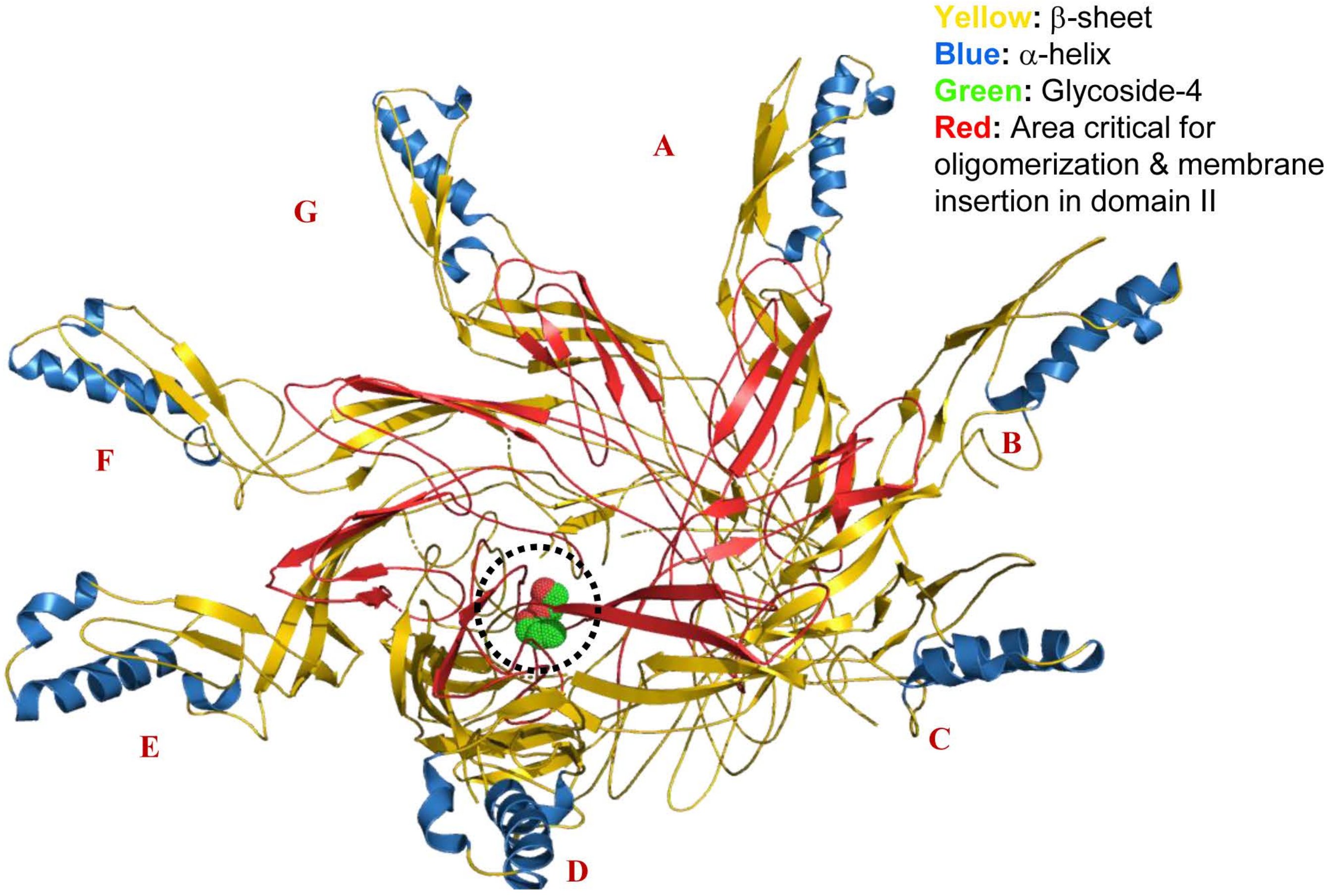

Fig S3: *In vitro* and *in silico* screening of glycosides against Etx. A) All the glycosides were screened at 20 μM and 50 μM concentrations against Etx. One-way ANOVA was used to compare the difference using post-hoc (Bonferroni) test. Statistical significance (P<0.005, P<0.01, P<0.05 vs. Etx) is shown. B) Cartoon representation of ligand-bound Etx showing the structures within region of maximum binding.

Disease Models & Mechanisms • Supplementary information

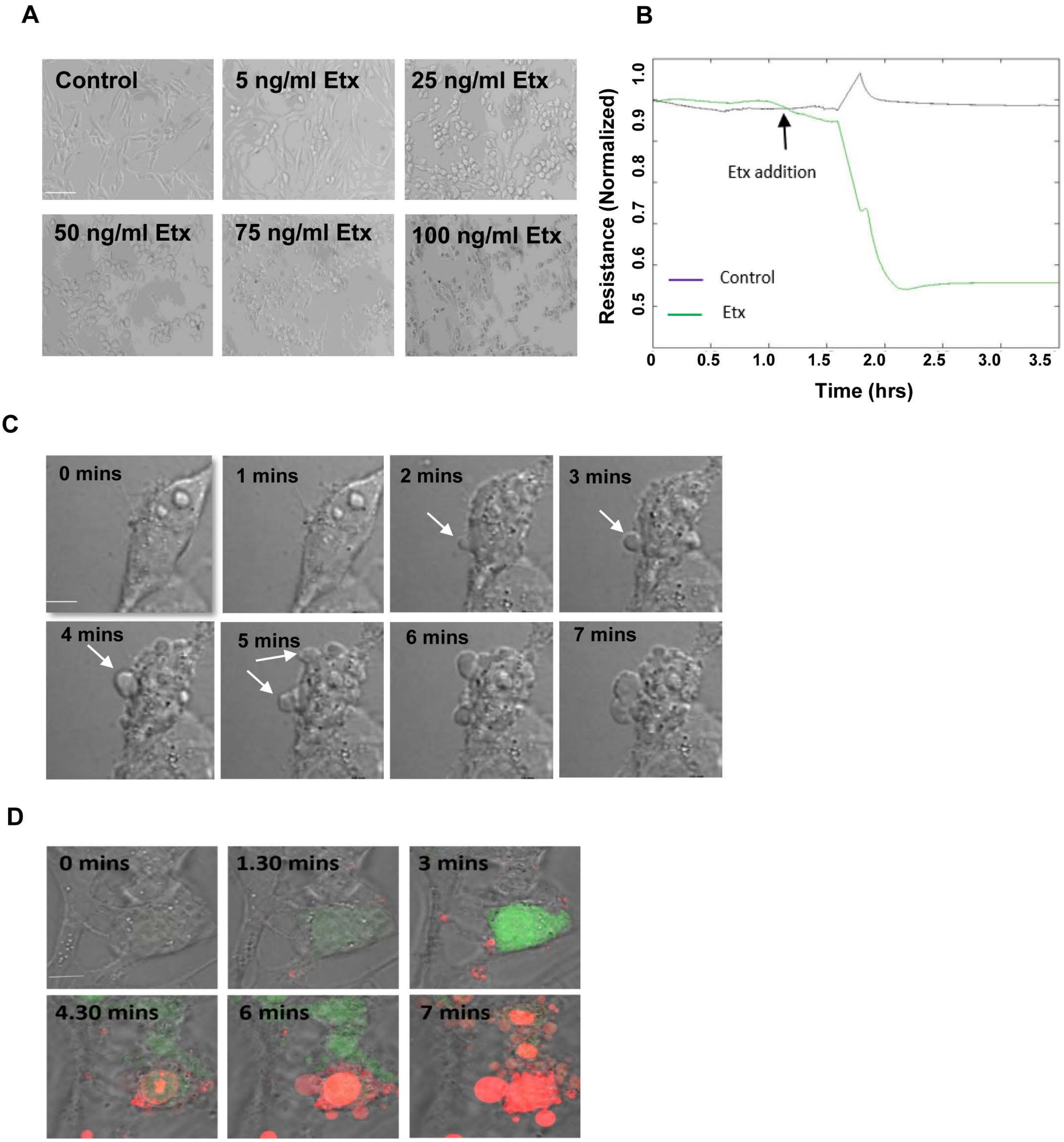

Disease Models & Mechanisms • Supplementary information

Fig S4: Purified Etx evokes necrotic death of MDCK cells *in vitro*. A) Microscopic analysis of the effect of Etx on cells. Indicated amount of Etx incubated with cells and microscopic analysis was performed. Control cells were incubated with media only. B) ECIS was performed to determine the changes in TEER. Upon Etx treatment a decrease in TEER was observed compared to the control cells. C) MDCK cells were treated with Etx and time lapse video microscopy was performed. Selected image frames are represented. D) Intracellular Ca^{2+} levels were monitored in Etx treated cells loaded with Fluo-4AM (green) by live cell microscopy. An increase Ca^{2+} was seen just before the PI (red) positivity. Selected frames of Fluo-4AM and PI merged with DIC are represented. Scale bar indicated 5 μM.

Table S1: The structures of all the glycosides (1-12) are depicted.

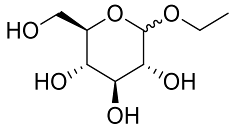 **Glycoside-1**	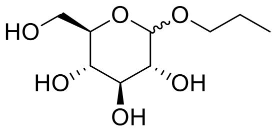 **Glycoside-2**	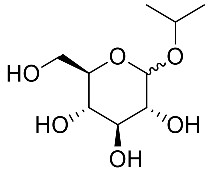 **Glycoside-3**	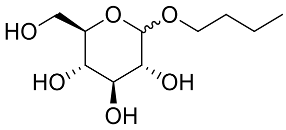 **Glycoside-4**
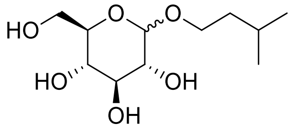 **Glycoside-5**	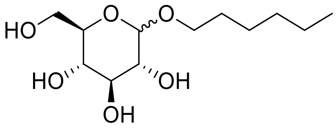 **Glycoside-6**	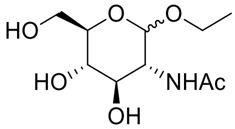 **Glycoside-7**	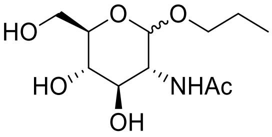 **Glycoside-8**
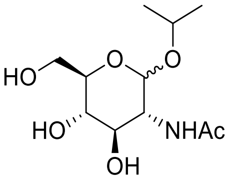 **Glycoside-9**	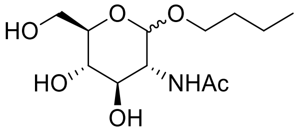 **Glycoside-10**	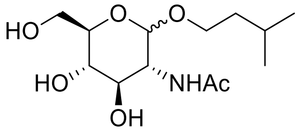 **Glycoside-11**	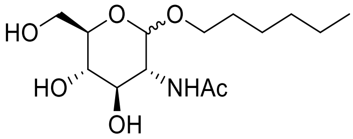 **Glycoside-12**

Disease Models & Mechanisms • Supplementary information

Table S2: Residues lying within the heptameric structure of Etx were found to form salt bridges within a close distance of 2.7-3.4 Å.

Salt bridges		
Residue 1	Residue 2	Distance
NZ LYS A 87	OD1 ASP B 250	2.74
NZ LYS A 87	OD1 ASP B 250	2.74
NZ LYS A 87	OD2 ASP B 250	2.71
NZ LYS B 87	OD1 ASP C 250	2.8
NZ LYS B 87	OD2 ASP C 250	2.75
NZ LYS B 108	OE1 GLU C 159	2.72
NZ LYS C 87	OD1 ASP D 250	2.84
NZ LYS C 87	OD2 ASP D 250	2.69
NZ LYS C 108	OE1 GLU D 159	3.15
NZ LYS D 87	OD1 ASP E 250	2.75
NZ LYS D 87	OD2 ASP E 250	2.95
NZ LYS D 108	OE1 GLU E 159	2.73
NZ LYS E 87	OD2 ASP F 250	2.68
NZ LYS E 108	OE1 GLU F 159	2.8
NH1 ARG E 257	OD2 ASP F 250	3.46
NZ LYS F 87	OD1 ASP G 250	2.74
NZ LYS F 108	OE1 GLU G 159	2.86
NH1 ARG F 257	OE1 GLU G 74	2.72
NH2 ARG F 257	OE1 GLU G 74	2.8
NZ LYS G 184	OE1 GLU F 178	2.9
NZ LYS G 87	OD1 ASP A 250	2.78
NZ LYS G 87	OD2 ASP A 250	2.79
NZ LYS G 108	OE1 GLU A 159	2.77
NH1 ARG G 257	OD1 ASP A 250	2.7
NH2 ARG G 257	OD1 ASP A 250	3.71

Disease Models & Mechanisms • Supplementary information

Table S3: Critical residues lying within the docked ligand-protein complex were found to be forming H-bonds ranging from 2.6 to 3.3 Å.

**Hydrogen bonding**		
Residue 1	Residue 2	Distance
O Glu E 159	O5 Glycoside-4	3.07
OG1 Thr D 106	O5 Glycoside-4	3.12
OG1 Thr D 106	O6 Glycoside-4	2.69
OG1 Thr D 106	O1 Glycoside-4	3.30
N Cys D 107	O1 Glycoside-4	3.25
O Val D 164	O3 Glycoside-4	3.19
O Asn D 163	O3 Glycoside-4	2.97
O Thr E 161	O4 Glycoside-4	3.34
O Thr E 161	O6 Glycoside-4	2.62
OG1 Thr E 161	O6 Glycoside-4	3.03

Disease Models & Mechanisms • Supplementary information

Text. S1

S1. A (3*R*,4*S*,5*S*,6*R*)-2-ethoxy-6-(hydroxymethyl) tetrahydro-2*H*-pyran-3,4,5-triol

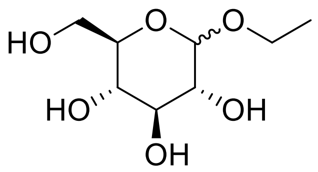

Glycoside-1

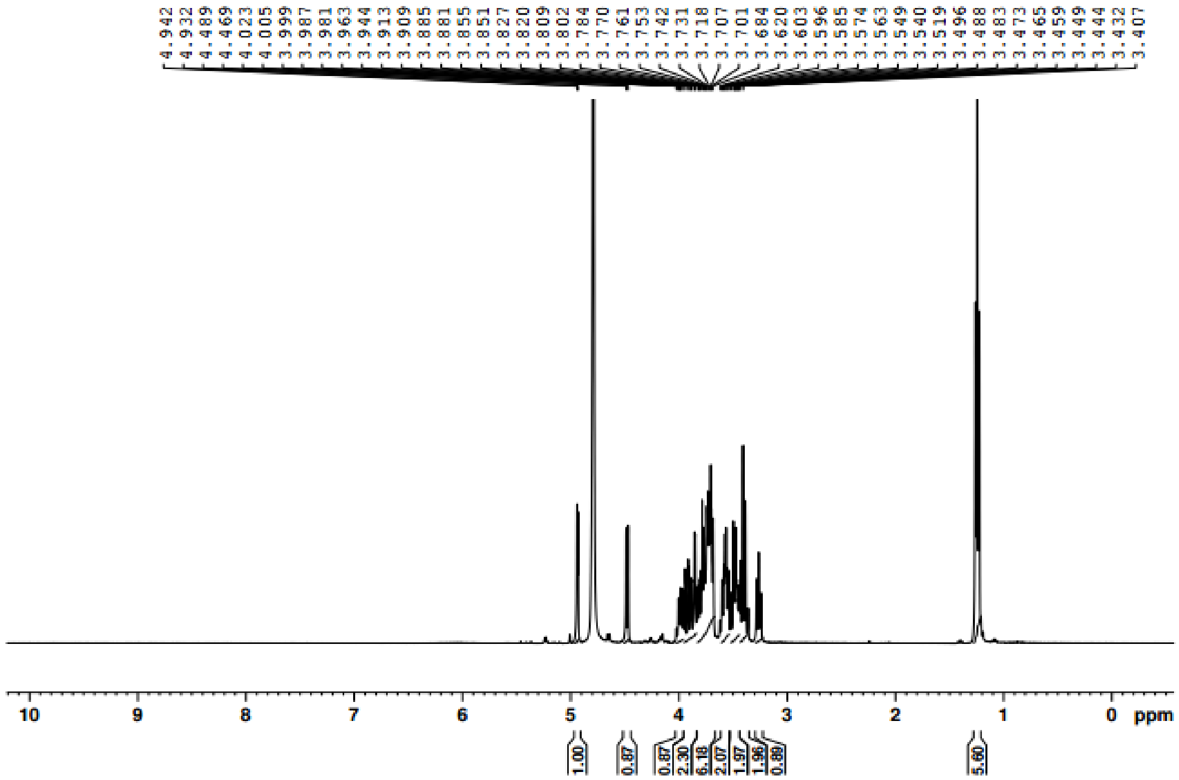

Disease Models & Mechanisms • Supplementary information

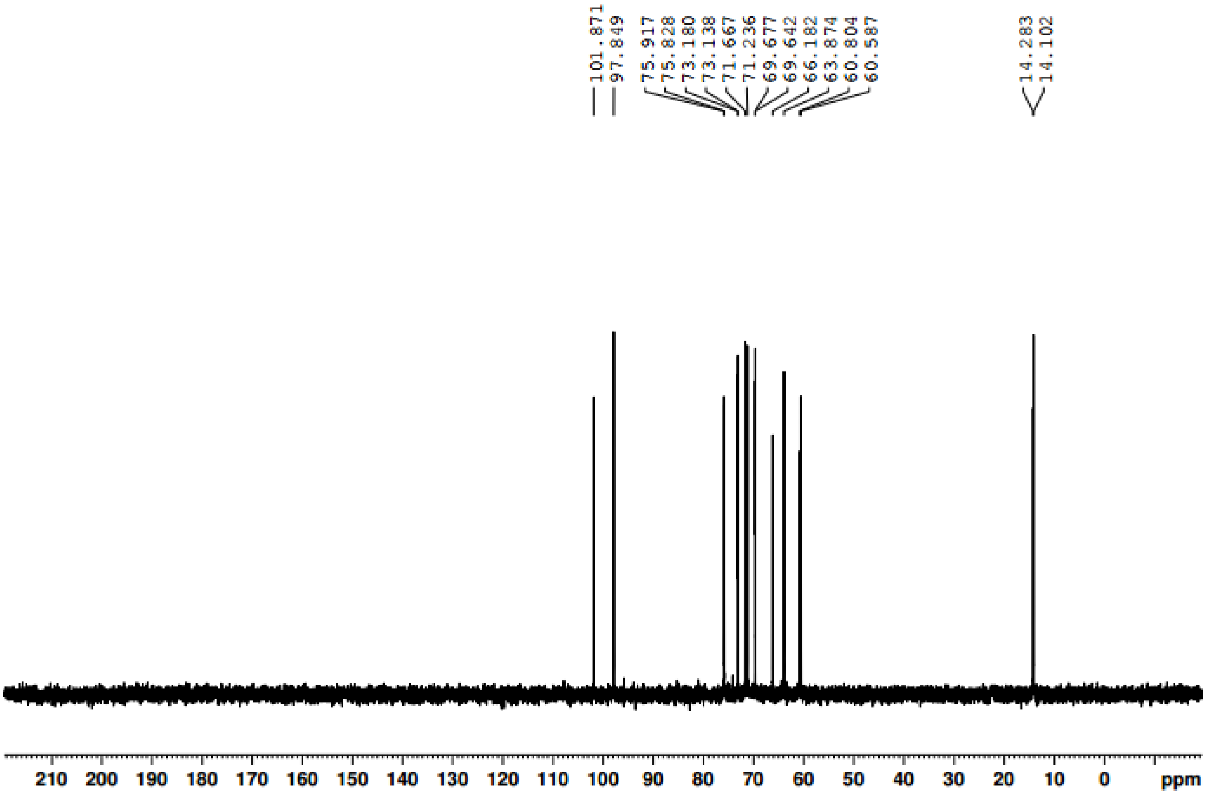

S1. A (3*R*,4*S*,5*S*,6*R*)-2-ethoxy-6-(hydroxymethyl) tetrahydro-2*H*-pyran-3,4,5-triol

The title compound **Glycoside-1** was isolated as amorphous solid (60%), anomeric ratio (α : β, 54 : 46); ^{1}H NMR (400 MHz, D_2O): δ 4.93 (d, *J* = 3.6 Hz, 1H, H-1β), 4.47 (d, *J* = 8 Hz, 0.44H, H-1α), 3.98 (dd, *J* = 2.4 Hz, 7.2 Hz, 1H), 3.92 (dd, *J* = 12.4 Hz, 1.6 Hz, 1H), 3.87 (dd, *J* = 1.6 Hz, 12 Hz, 1H), 3.82-3.76 (m, 2H), 3.75-3.68 (m, 4H), 3.61-3.53 (m, 2H), 3.49-3.44 (m, 2H), 3.39 (dd, *J* = 10 Hz, 19.2 Hz, 2H), 3.26 (t, *J* = 8.4 Hz, 1H), 1.24 (t, *J* = 7.2 Hz, 3H); ^{13}C NMR (100 MHz, D_2O): δ 101.8 (C-1β), 97.8 (C-1α), 75.9, 75.8, 73.1, 73.1, 71.6, 69.6, 69.6 (C-3, C-4, C-5), 66.1, 63.8, 60.8, 60.5, 14.2, 14.1; HRMS (ESI) *m/z* calcd for $C_8H_{16}O_6$ $[M+Na]^+$ 231.0839, found 231.0870.

S1. B (2*R*,3*S*,4*S*,5*R*)-2-(hydroxymethyl)-6-propoxytetrahydro-2*H*-pyran-3,4,5-triol

Glycoside-2

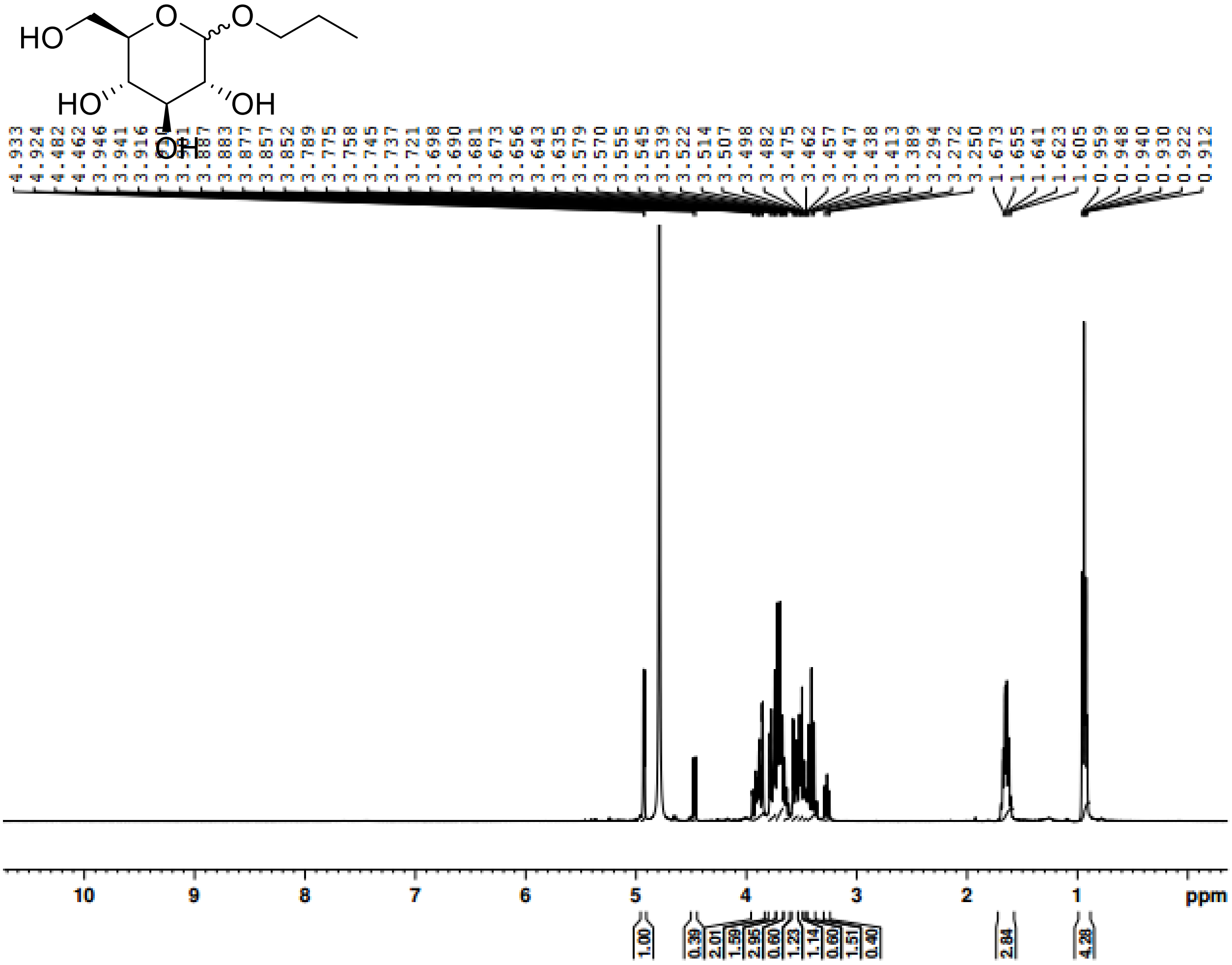

Disease Models & Mechanisms • Supplementary information

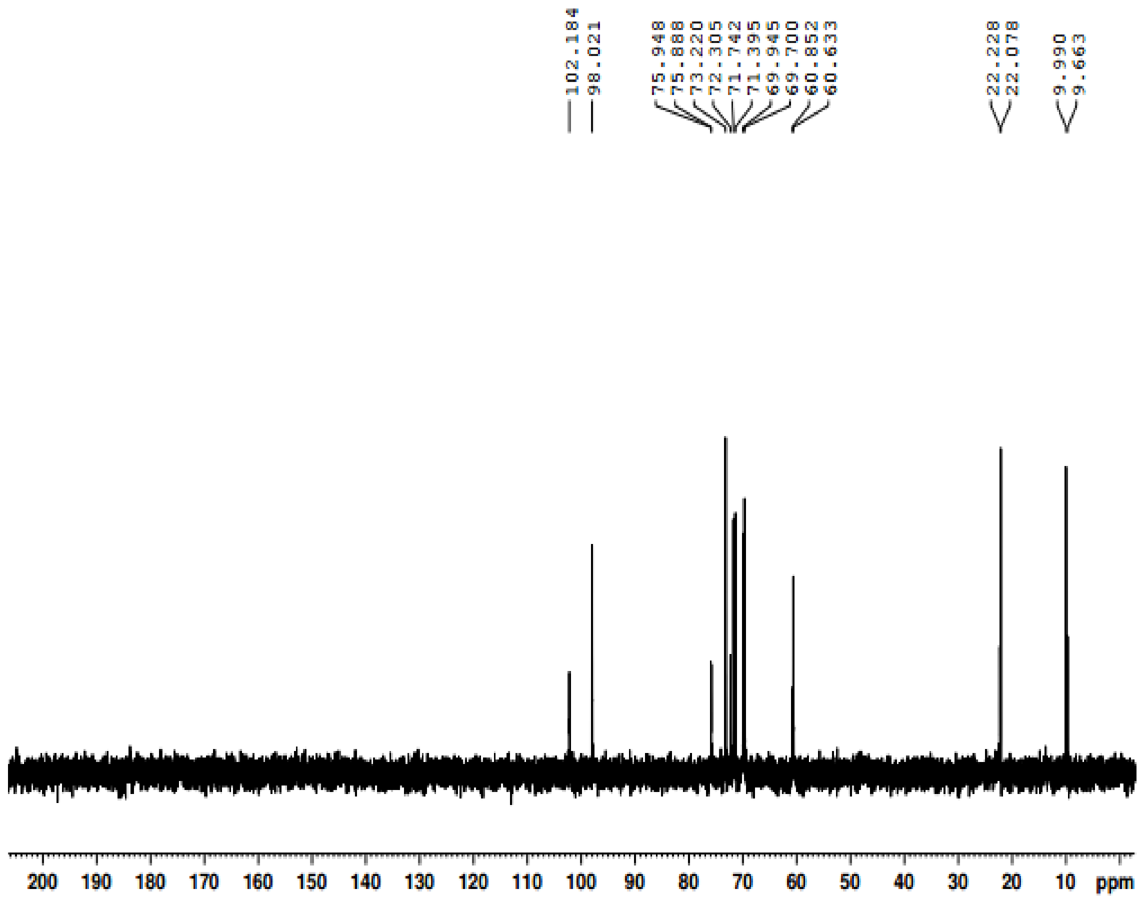

S1. B (2*R*,3*S*,4*S*,5*R*)-2-(hydroxymethyl)-6-propoxytetrahydro-2*H*-pyran-3,4,5-triol

The title compound was purified by column chromatography (EtOAc/MeOH = 95:5) furnished compound **Glycoside-2** as sticky solid 0.19 mg (77 %), anomeric ratio (α: β = 7:3); ^{1}H NMR (400 MHz, D_2O) δ 4.93 (d, *J* = 3.6 Hz, 1H, H-1β), 4.47 (d, *J* = 8.0 Hz, 1H), 3.94-3.85 (m, 2H), 3.78-3.73 (m, 2H), 3.78-3.73 (m, 1.5H), 3.73-3.65 (m, 3H), 3.56 (dd, *J* = 3.6 Hz, 9.6 Hz, 1H), 3.50 (dd, *J* = 3.2 Hz, 6Hz, 1H), 3.41 (t, *J* = 10 Hz, 1H), 3.27 (t, *J* = 8.8 Hz, 1H), 1.65 (quin, *J* = 7.2 Hz, 3H), 0.94 (t, *J* = 7.2 Hz, 4H); ^{13}C NMR (100 MHz, D_2O) δ 102.1 (C-1β), 98.0 (C-1α), 75.9, 75.8, 73.2, 72.3, 71.7, 71.3, 69.9, 69.7, 60.8, 60.6, 22.2, 22.0, 93.9.9, 9.6; HRMS(ESI) *m/z* calcd for $C_9H_{18}O_6$ [M+Na]$^+$ 245.0996, found 245.1024.

Disease Models & Mechanisms • Supplementary information

S1. C (2*R*,3*S*,4*S*,5*R*)-2-(hydroxymethyl)-6-isopropoxytetrahydro-2*H*-pyran-3,4,5-triol

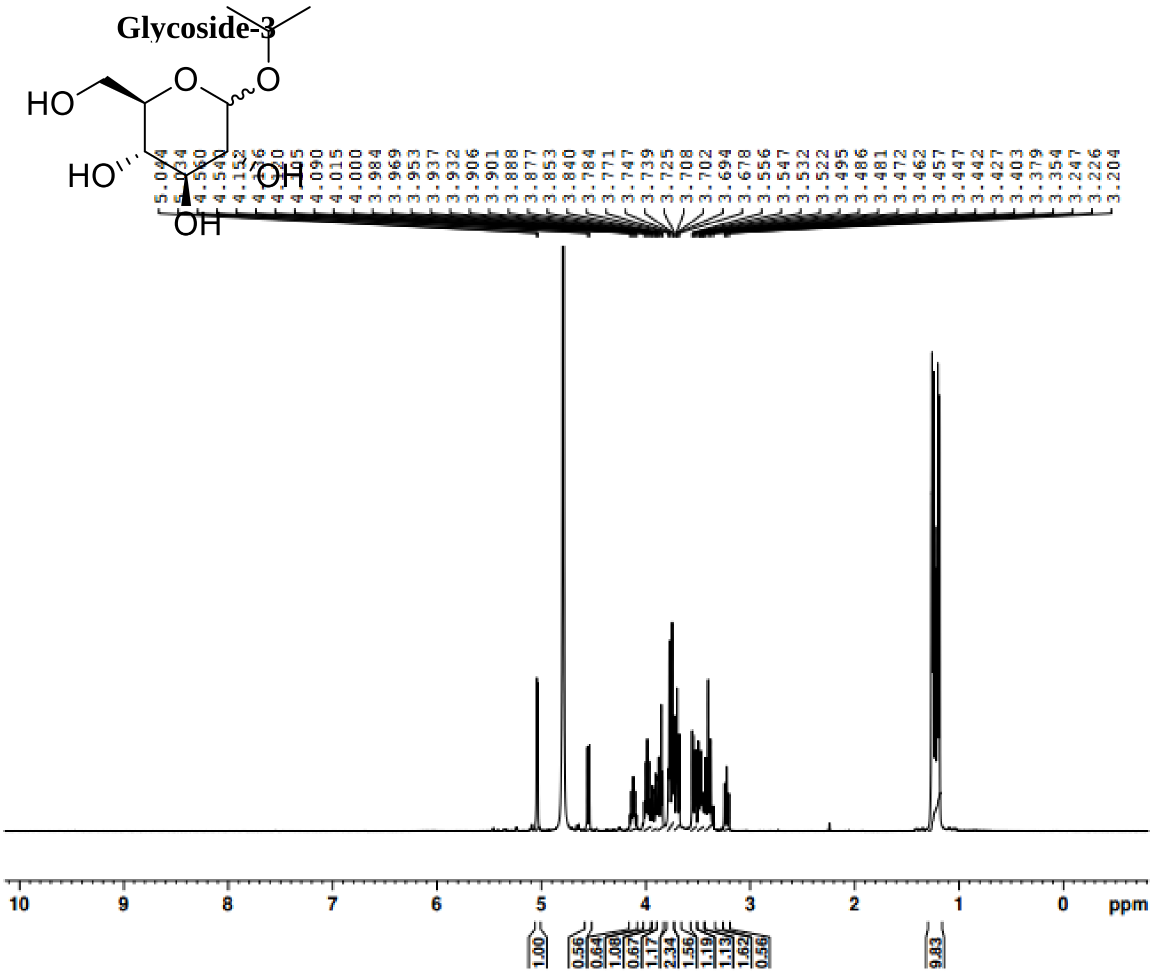

Disease Models & Mechanisms • Supplementary information

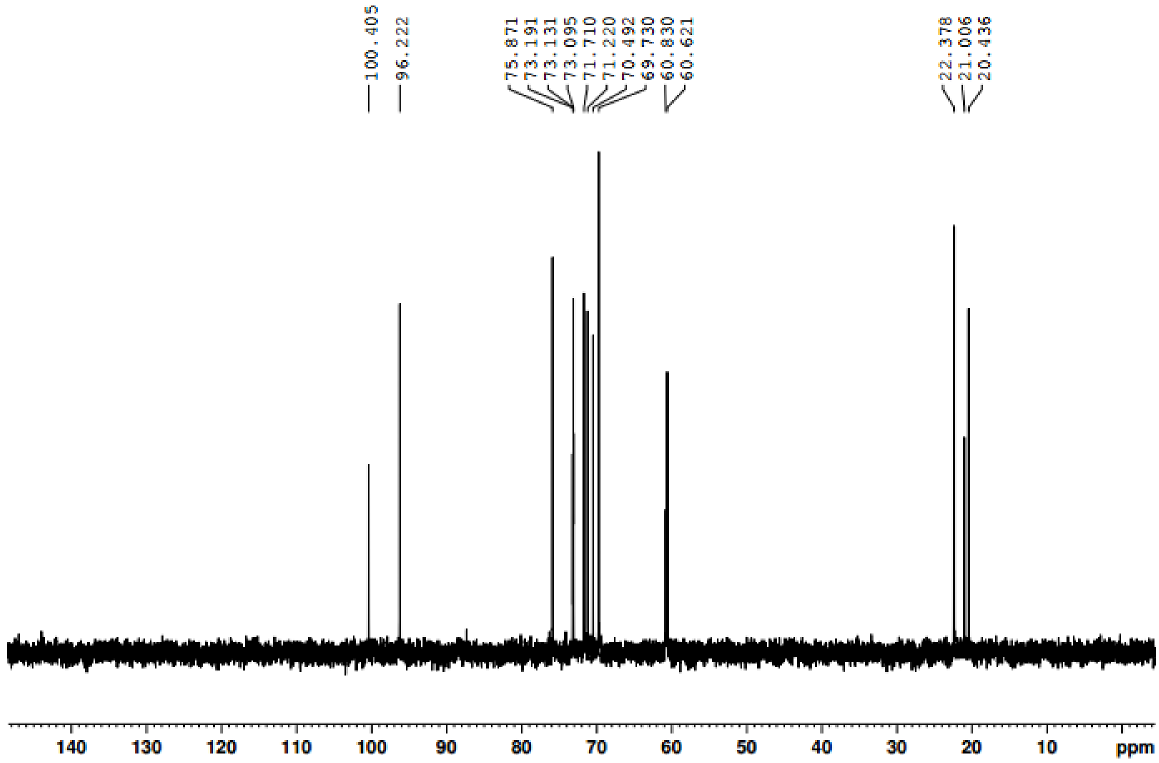

S1. C (2*R*,3*S*,4*S*,5*R*)-2-(hydroxymethyl)-6-isopropoxytetrahydro-2*H*-pyran-3,4,5-triol

The title compound was purified by column chromatography (EtOAc/MeOH = 95:5) furnished compound **Glycoside-3** as yellow colouroil 0.12g (60%), anomeric ratio (α:β, 65:35): ^{1}H NMR (400 MHz, D_2O**)** δ 5.03 (d, *J* = 3.6 Hz, 1H, H-1β), 4.55 (d, *J* = 8 Hz, 0.5H, H-1α), 4.12 (quin, *J* = 6 Hz, 1H), 3.98 (quin, *J* = 6.4 Hz, 1H), 3.92 (dd, *J* = 2 Hz, 12 Hz, 1H), 3.86 (dd, *J* = 4.4 Hz, 14.4 Hz, 1H), 3.75 (dd, *J* = 5.2 Hz, 14.8 Hz, 2H), 3.70 (dd, *J* = 6.4 Hz, 2.8 Hz, 1H), 3.54 (dd, *J* = 3.6 Hz, 9.6 Hz, 1H), 3.49-3.44 (m, 1H), 3.38 (t, *J* = 9.6 Hz, 1H), 3.22 (t, *J* = 8.4 Hz, 1H), 1.24 (d, J = 6 Hz, 2H), 1.21 (d, *J* = 6 Hz, 3H), 1.19 (t, *J* = 6.0 Hz, 3H)**;** ^{13}C NMR (100 MHz, D_2O): δ 100.4 (C-1β), 96.2 (C-1α), 75.8, 73.2, 73.1, 73.0, 71.7, 71.2, 70.4, 69.7, 60.8, 60.6, 22.3, 21.0, 20.4; HRMS(ESI) m/z calcd $C_9H_{18}O_6$, $[M+Na]^+$ 245.0996, found 245.1026.

Disease Models & Mechanisms • Supplementary information

S1. D (3*R*,4*S*,5*S*,6*R*)-2-butoxy-6-(hydroxymethyl) tetrahydro-2*H*-pyran-3,4,5-triol

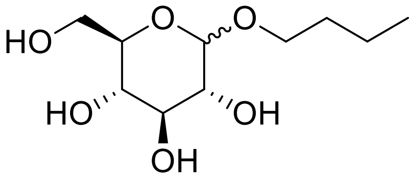

Glycoside-4

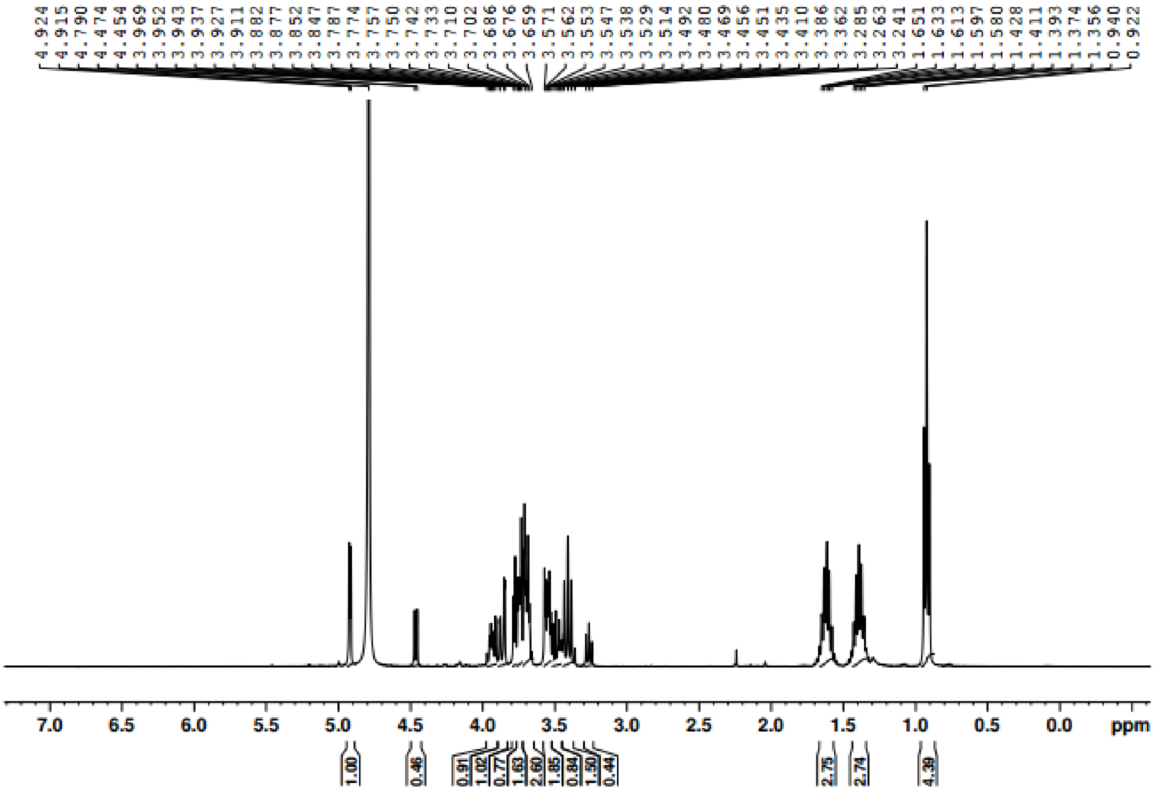

Disease Models & Mechanisms • Supplementary information

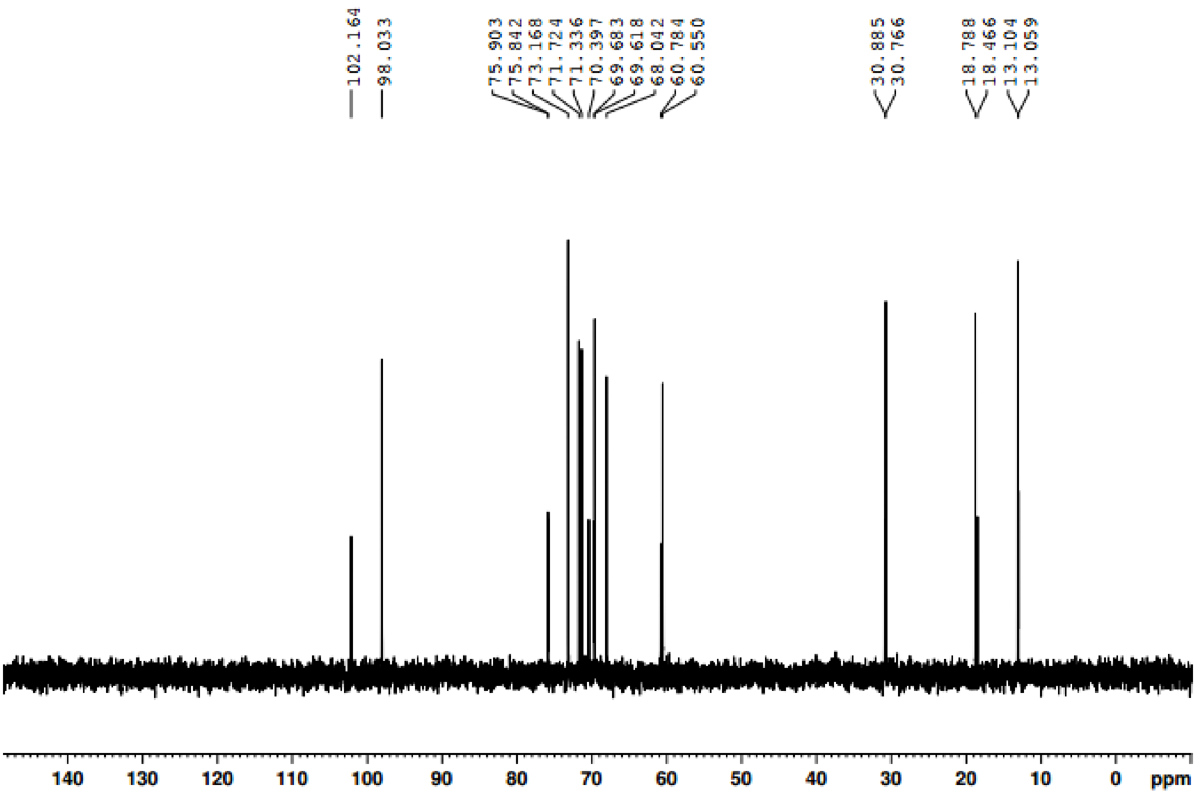

S1. D (3*R*,4*S*,5*S*,6*R*)-2-butoxy-6-(hydroxymethyl) tetrahydro-2*H*-pyran-3,4,5-triol

The title compound was isolated by column chromatography (CH_2Cl_2/MeOH = 95:5) furnished compound **Glycoside-4** as yellow color oil 0.2 g (76%), anomeric ratio (α:β, 7:3); ^{1}H NMR (400 MHz, D_2O): δ 4.91 (d, *J* = 3.6 Hz, 1H, H-1α), 4.46 (d, *J* = 8 Hz, 0.44H, H-1β), 3.91 (m,1H), 3.86 (dd, *J* = 2.0 Hz, 12.4 Hz, 1H), 3.78 (d, *J* = 4.8 Hz, 1H), 3.74 (dd, *J* = 3.2 Hz, 6 Hz, 1H), 3.70-3.67 (m, 2H), 3.54 (dt, *J* = 3.6 Hz, 7.6 Hz, 2H), 3.41 (t, *J* = 9.6 Hz, 2H), 3.26 (t, *J* = 8.4 Hz, 0.4H, H-4), 1.61 (m, 3H), 1.39 (m, 3H), 0.92 (t, *J* = 7.6 Hz, 4H); ^{13}C NMR (100 MHz, D_2O): δ 102.16 (C-1β), 98.03 (C-1α), 75.9, 75.8, 73.1, 71.7, 71.3, 70.3 (C-3, C-4, C-5), 69.6, 69.6, 68.0 (C-6), 60.7, 60.5, 30.8, 30.7, 18.7, 18.4, 13.1, 13.0; HRMS (ESI) *m/z* calcd for $C_{10}H_{20}O_6$, $[M+Na]^+$: 259.1152, found 259.1189.

Disease Models & Mechanisms • Supplementary information

S1. E (2*R*,3*S*,4*S*,5*R*)-2-(hydroxymethyl)-6-(isopentyloxy) tetrahydro-2*H*-pyran-3,4,5-triol

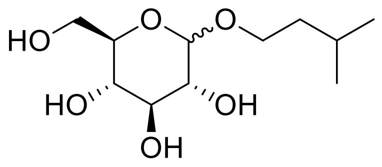

Glycoside-5

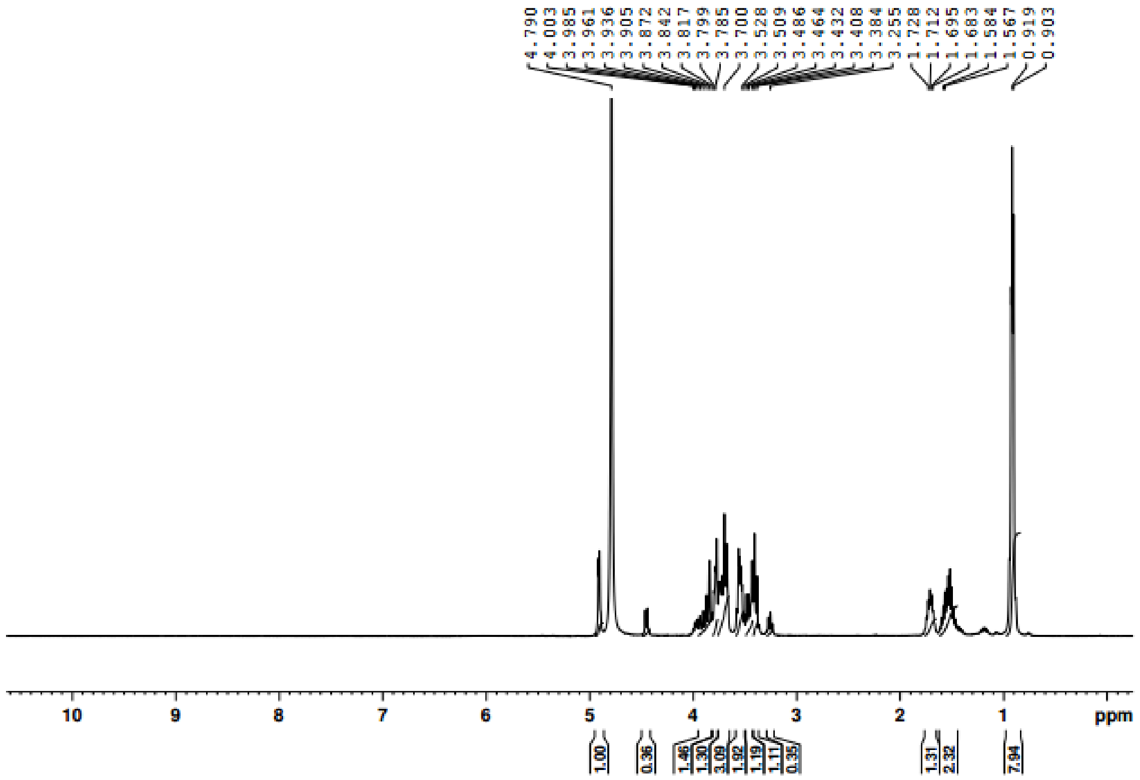

Disease Models & Mechanisms • Supplementary information

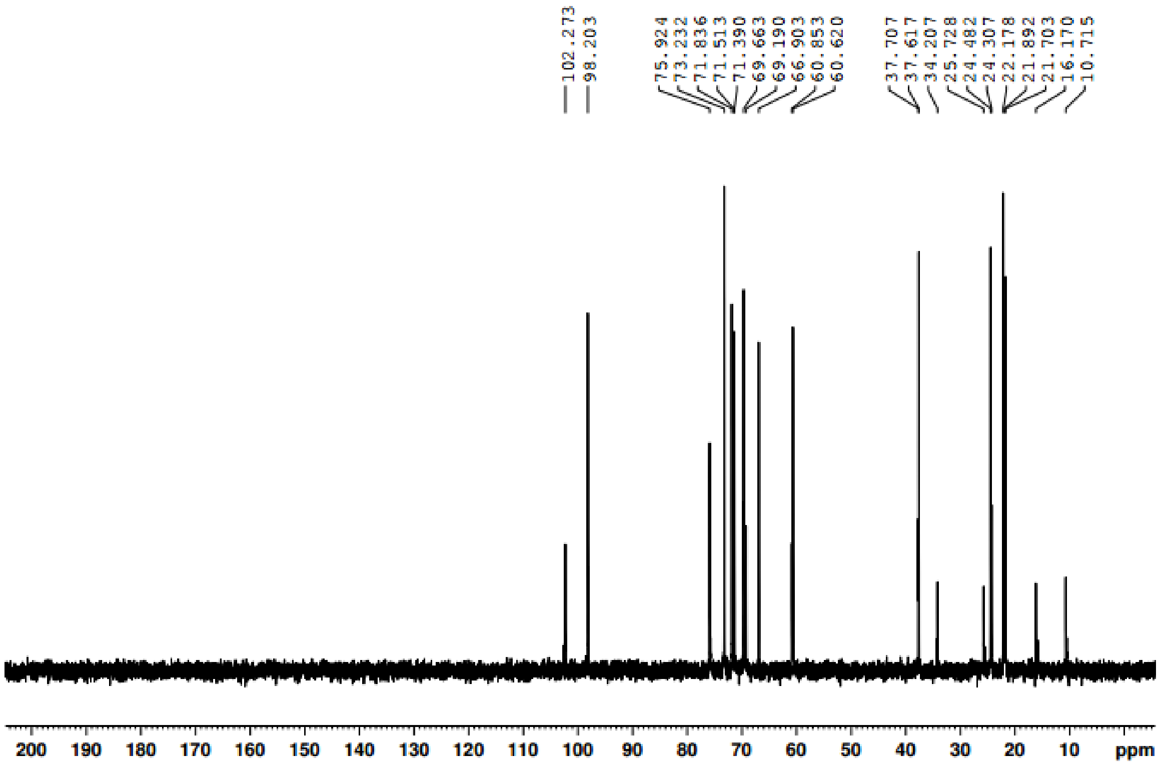

S1. E (2*R*,3*S*,4*S*,5*R*)-2-(hydroxymethyl)-6-(isopentyloxy) tetrahydro-2*H*-pyran-3,4,5-triol

The title compound was isolated by column chromatography (EtOAc/MeOH = 95:5) furnished compound **Glycoside-5** as sticky solid 0.1 g (62.5%), anomeric ratio (α: β = 74 : 26); ^{1}H NMR (400 MHz, D_2O): δ 4.91 (d, *J* = 4 Hz, 1H, H-1β), 4.45 (d, *J* = 8Hz, 1H), 4.00-3.87 (m, 1H), 3.84-3.77 (m, 1H), 3.74-3.67 (m, 3H), 3.58-3.52 (m, 2H), 3.48 (dd, *J* = 7.6 Hz, 1H), 3.40 (t, *J* = 9.6 Hz, 1H), 3.25 (t, *J* = 8.4 Hz, 1H), 1.74-1.68 (m, 1.3H), 1.61-1.46 (m, 2H), 0.92-0.90 (m, 7H); ^{13}C NMR (100 MHz, D_2O): δ 102.1 (C-1β), 98.0 (C-1α), 75.92, 73.2, 71.8, 71.5, 71.3, 69.6, 69.1, 66.9, 60.8, 60.6, 37.7, 37., 34.2, 25.7, 24.4, 24.3, 22.1, 21.8, 21.7, 16.17, 10.7; HRMS (ESI) *m/z* calcd for $C_{11}H_{22}O_6$, $[M+Na]^+$: 273.1309, found 273.1342.

S1. F (3*R*,4*S*,5*S*,6*R*)-2-(hexyloxy)-6-(hydroxymethyl) tetrahydro-2*H*-pyran-3,4,5-triol

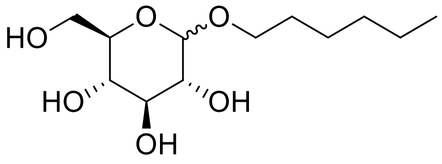

Glycoside-6

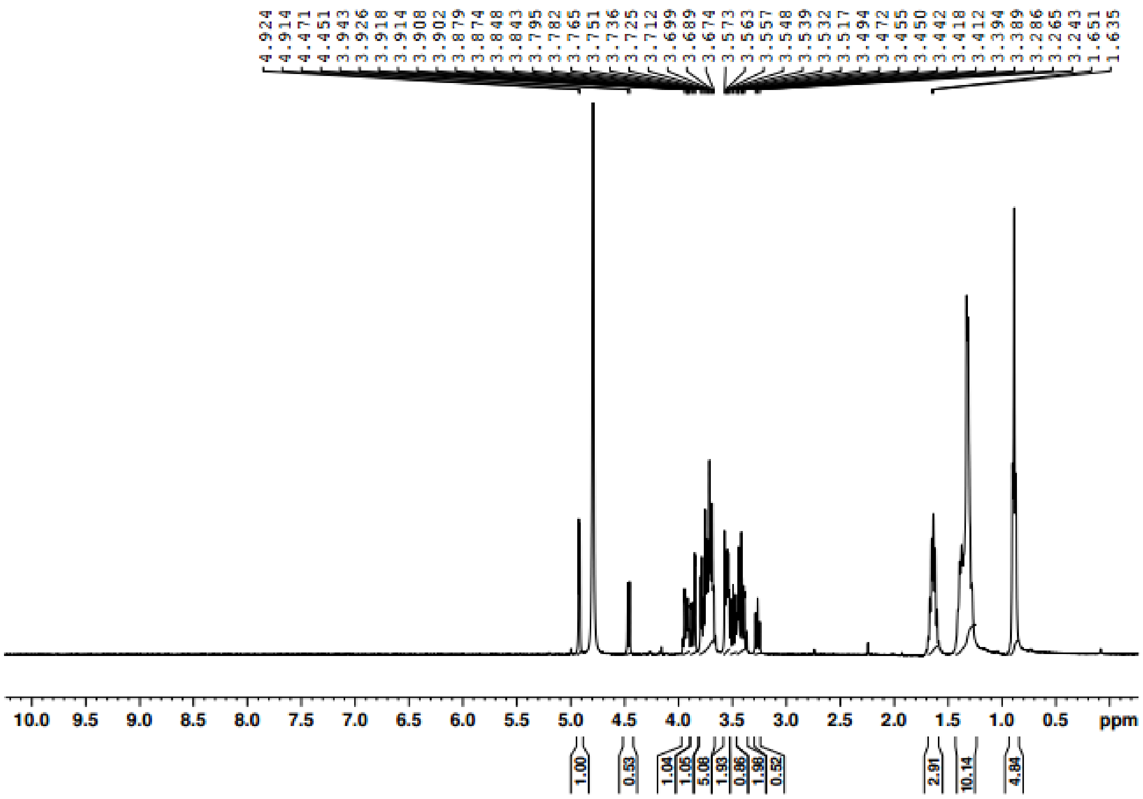

Disease Models & Mechanisms • Supplementary information

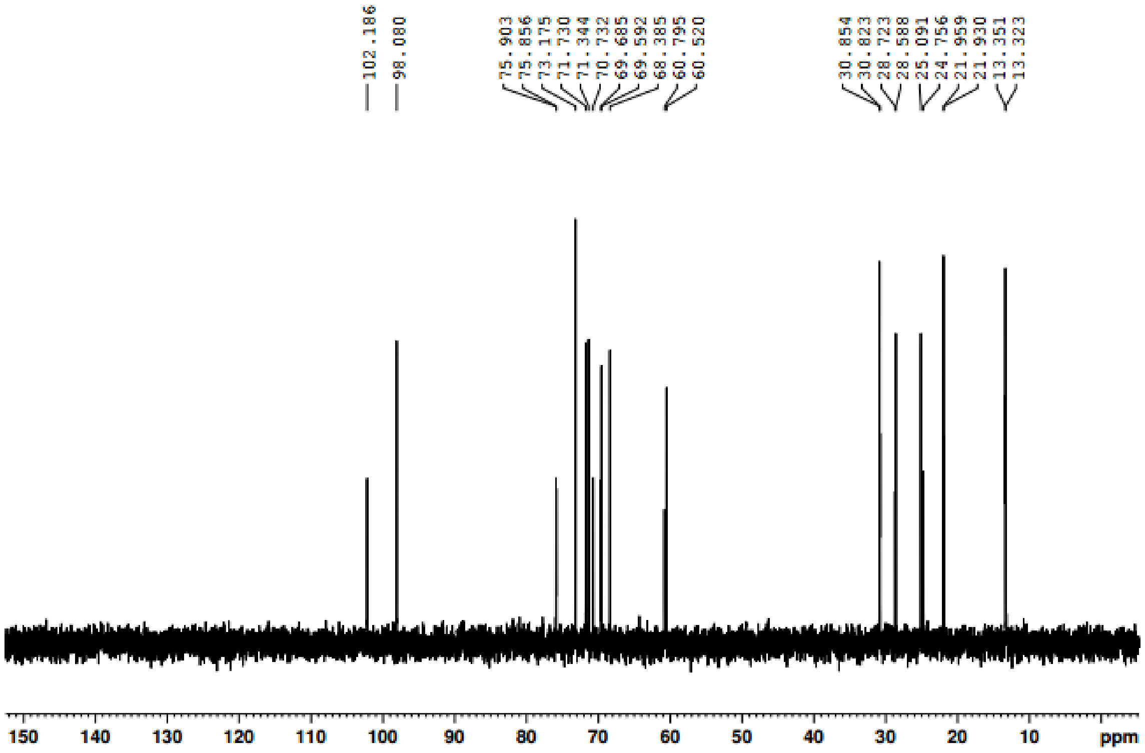

S1. F (3*R*,4*S*,5*S*,6*R*)-2-(hexyloxy)-6-(hydroxymethyl) tetrahydro-2*H*-pyran-3,4,5-triol

The title compound was isolated by column chromatography (EtOAc/MeOH = 95:5) furnished compound **Glycoside-6** as yellow colour sticky solid 0.13 g (44%), anomeric ratio (α:β, 2:1); ^{1}HNMR (400 MHz, D_2O): δ 4.91 (d, *J* = 4 Hz, 1H, H-1β), 4.46 (d, *J* = 8 Hz, 0.5H, H-1α), 3.94-3.90 (m, 1H), 3.85 (dd, *J* = 2 Hz, 12.4 Hz, 1H), 3.79-3.67 (m, 5H), 3.57-3.53 (m, 2H), 3.49 (t, *J* = 8.8 Hz, 1H), 3.43 (d, *J* = 9.2 Hz, 1H), 3.39 (dd, *J* = 6.8 Hz, 8.8 Hz, 1H), 3.26 (t, *J* = 6 Hz, 0.5H), 1.63 (quin, *J* = 6.4 Hz, 3H), 1.39-1.31 (m, 10H), 0.88 (t, *J* = 6.4 Hz, 4.8H); ^{13}C NMR (100 MHz, D_2O): δ 102.18 (C-1β), 98.08 (C-1α), 75.9, 75.8, 73.1, 71.7, 71.3, 70.7, 69.6, 69.5, 68.3, 60.7, 6.52, 30.85, 30.82, 28.72, 28.58, 25.09, 24.75, 21.95, 21.92, 13.35, 13.32; HRMS(ESI) m/z calcd for $C_{12}H_{24}O_6$ $[M+Na]^+$ 287.1465, found 287.1506.

S1. G *N*-((3*R*,4*R*,5*S*,6*R*)-2-ethoxy-4,5-dihydroxy-6-(hydroxymethyl) tetrahydro-2*H*-pyran-3-yl) acetamide

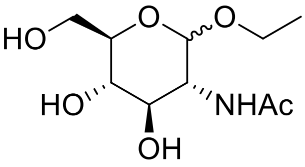

Glycoside-7

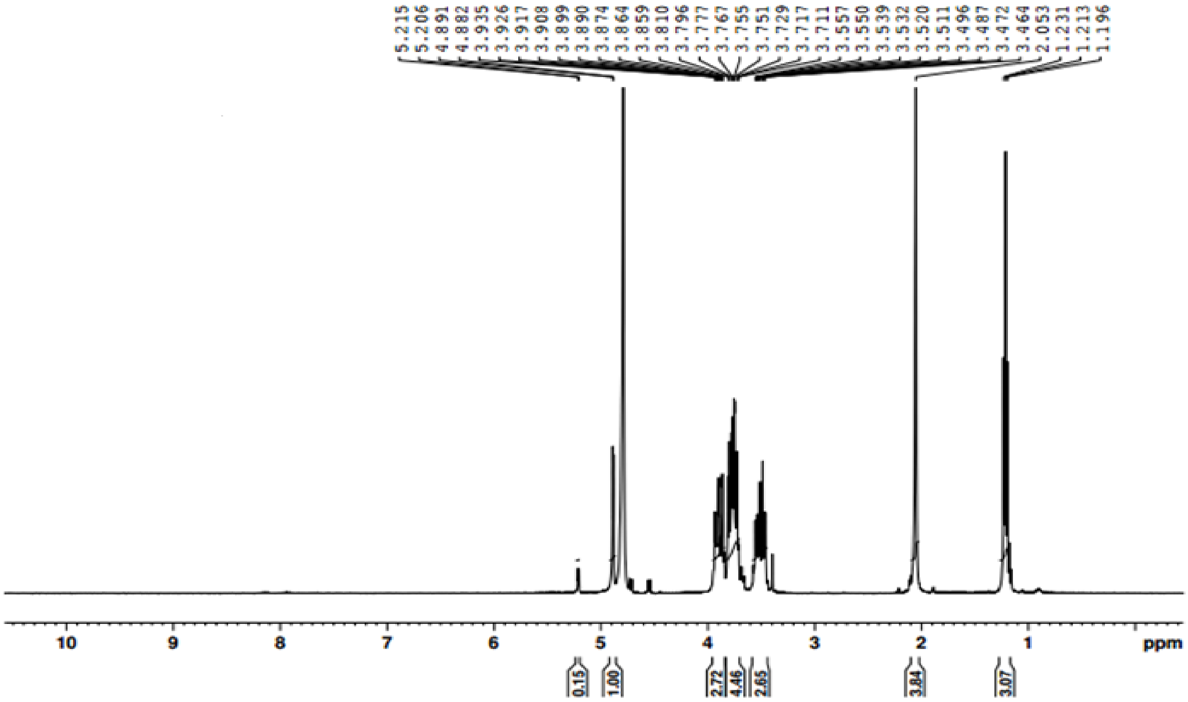

Disease Models & Mechanisms • Supplementary information

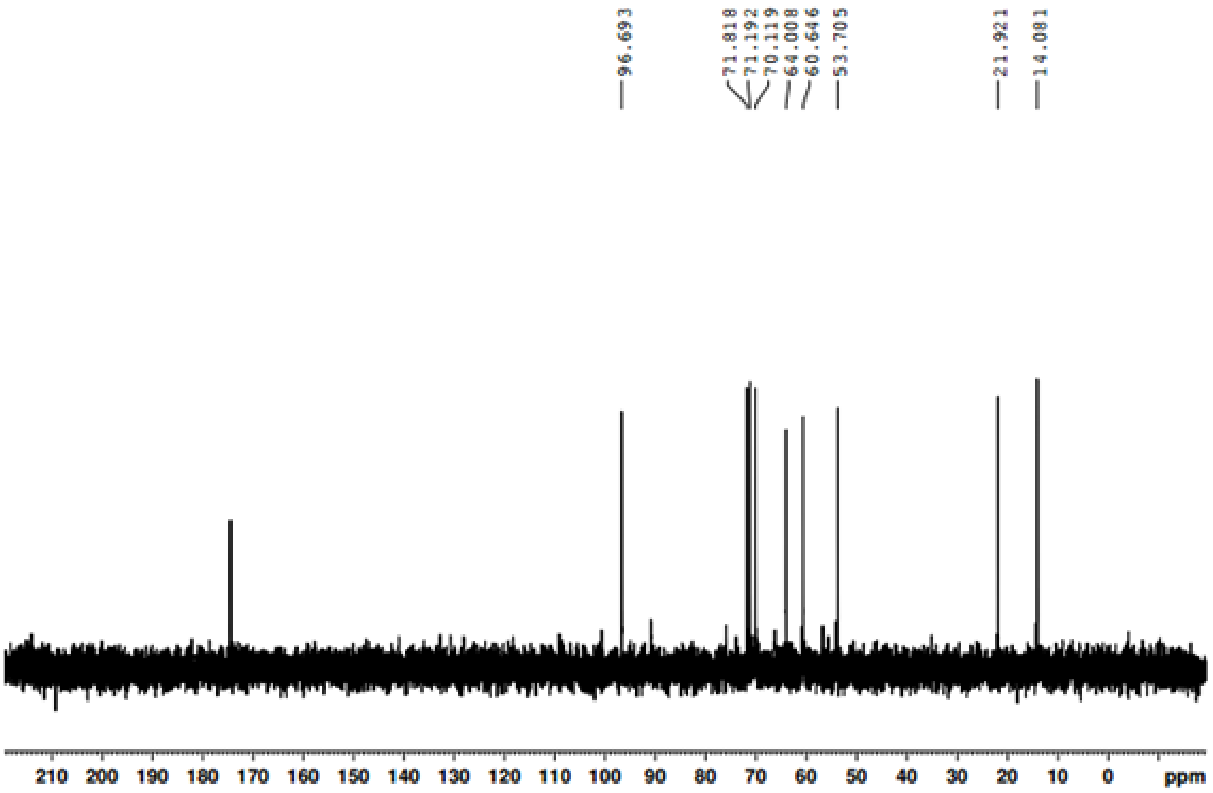

S1. G *N*-((3*R*,4*R*,5*S*,6*R*)-2-ethoxy-4,5-dihydroxy-6-(hydroxymethyl) tetrahydro-2*H*-pyran-3-yl) acetamide

The title compound **Glycoside-7** was isolated by column chromatography (EtOAc/MeOH = 95:5) in 41% yield. Compound was white color solid; anomeric ratio (α:β, 9:1); ^{1}H NMR (400 MHz, D_2O): δ 5.21 (d, *J* = 3.4 Hz, 0.14H, H-1α), 4.89 (d, *J* = 3.5 Hz, 1H, H-1β), 3.92-3.85 (m, 3H), 3.81-3.71 (m, 4H), 3.55-3.46 (m, 3H), 2.05 (s, 4H), 1.20 (t, *J* = 7.2 Hz, 3H); ^{13}C NMR (100 MHz, D_2O): δ 174.4 (C=O), 96.6 (C-1β), 71.8, 71.1, 70.1, 64.0, 60.6, 53.7, 21.9, 14.0, HRMS(ESI) *m/z* calcd for $C_{10}H_{19}NO_6$, $[M+Na]^+$ 272.1105, found 272.1139.

Disease Models & Mechanisms • Supplementary information

S1. H *N*-((3*R*,4*R*,5*S*,6*R*)-4,5-dihydroxy-6-(hydroxymethyl)-2-propoxytetrahydro-2H-pyran-3-yl)acetamide

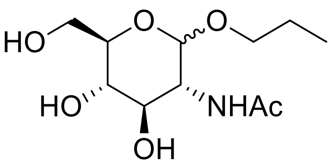

Glycoside-8

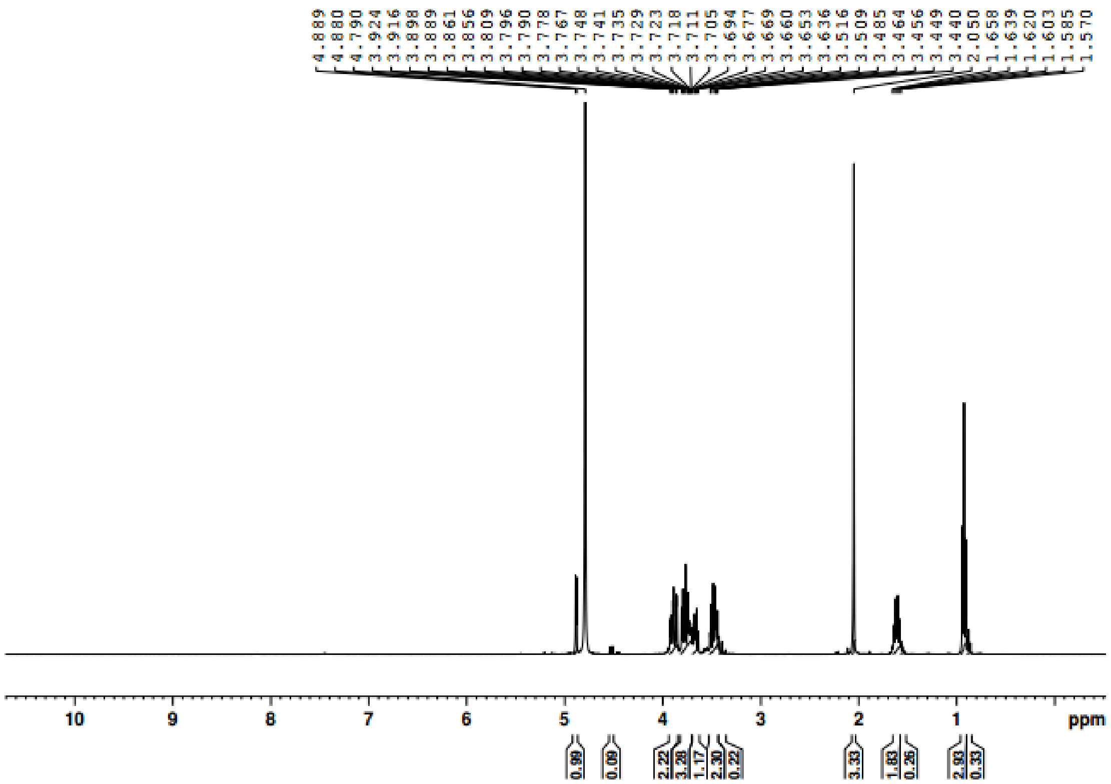

Disease Models & Mechanisms • Supplementary information

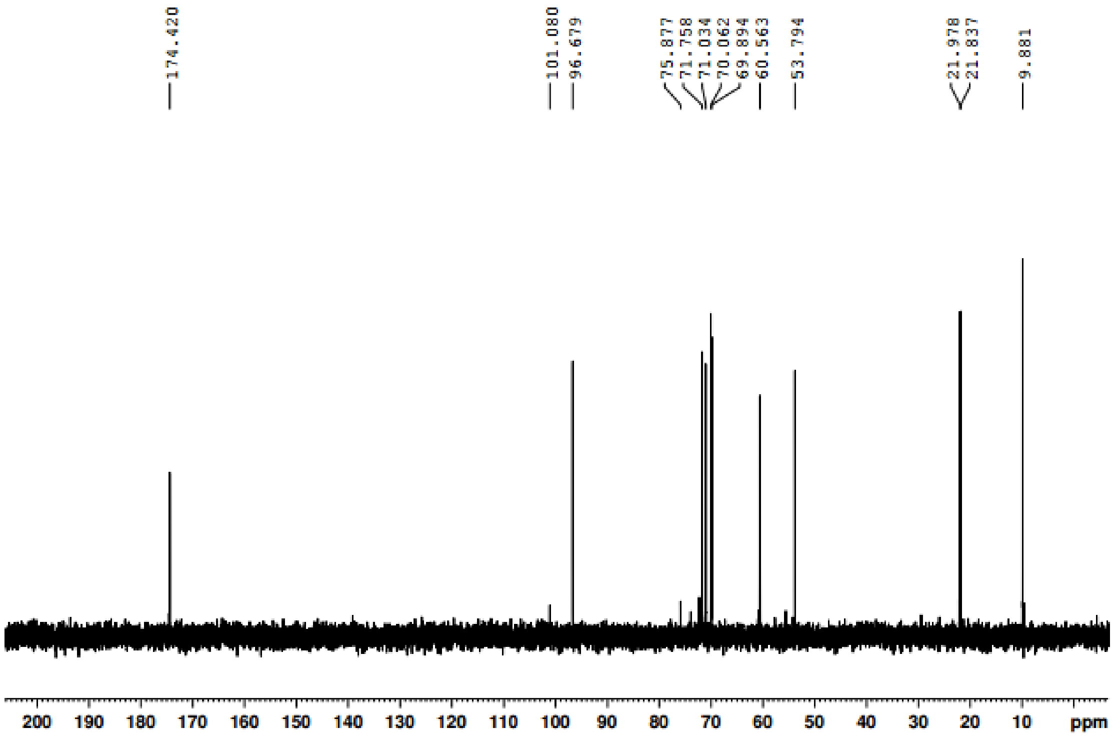

S1. H *N*-((3*R*,4*R*,5*S*,6*R*)-4,5-dihydroxy-6-(hydroxymethyl)-2-propoxytetrahydro-2H-pyran-3-yl)acetamide

The title compound was isolated by column chromatography (EtOAc/MeOH = 95:5) furnished compound **Glycoside-8** as white color compound 0.2 g (86 %), anomeric ratio (α:β, 92:8); ^{1}H NMR (400 MHz, CD_3OD): δ 4.88 (d, *J* = 3.6 Hz, 1H, H-1β), 3.88 (td, *J* = 3.6 Hz, 10.8 Hz, 2H), 3.80-3.70 (m, 3H), 3.66 (dt, *J* = 8 Hz, 10 Hz, 1H), 3.51-3.44 (m, 2H), 2.05 (s, 3H), 1.60 (s, *J* = 7.6 Hz, 2H), 0.92 (t, *J* = 7.2 Hz, 3H); ^{13}C NMR (100 MHz, D_2O): δ 174.4 (C=O), 101.0, 96.6 (C-1β), 75.8, 71.7, 71.0, 70.0, 69.8, 60.5, 53.7, 21.9, 21.8, 9.8; HRMS (ESI) *m/z* calcd for $C_{11}H_{21}NO_6$ $[M+Na]^+$ 286.1261, found 286.1299.

Disease Models & Mechanisms • Supplementary information

S1. I *N*-((3*R*,4*R*,5*S*,6*R*)-4,5-dihydroxy-6-(hydroxymethyl)-2-isopropoxytetrahydro-2*H*-pyran-3-yl) acetamide

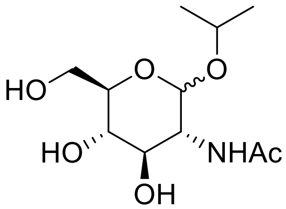

Glycoside-9

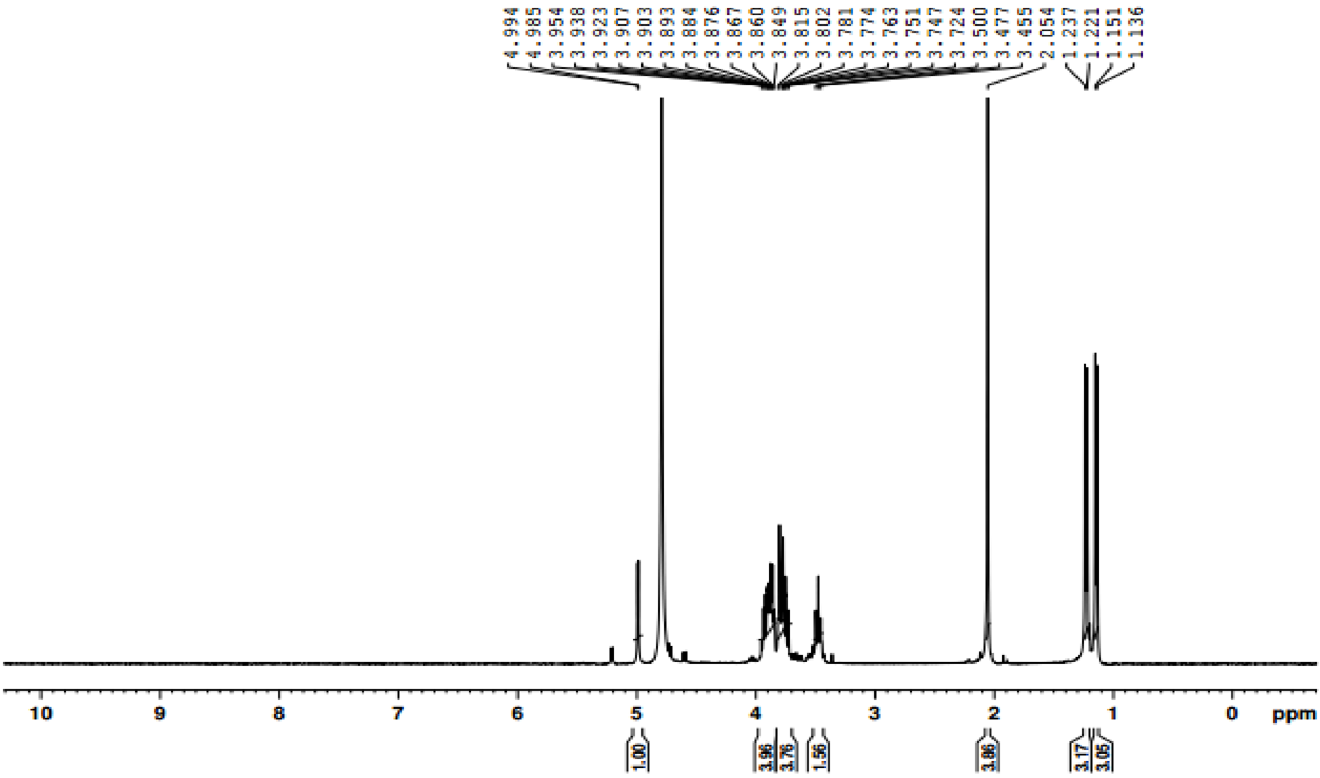

Disease Models & Mechanisms • Supplementary information

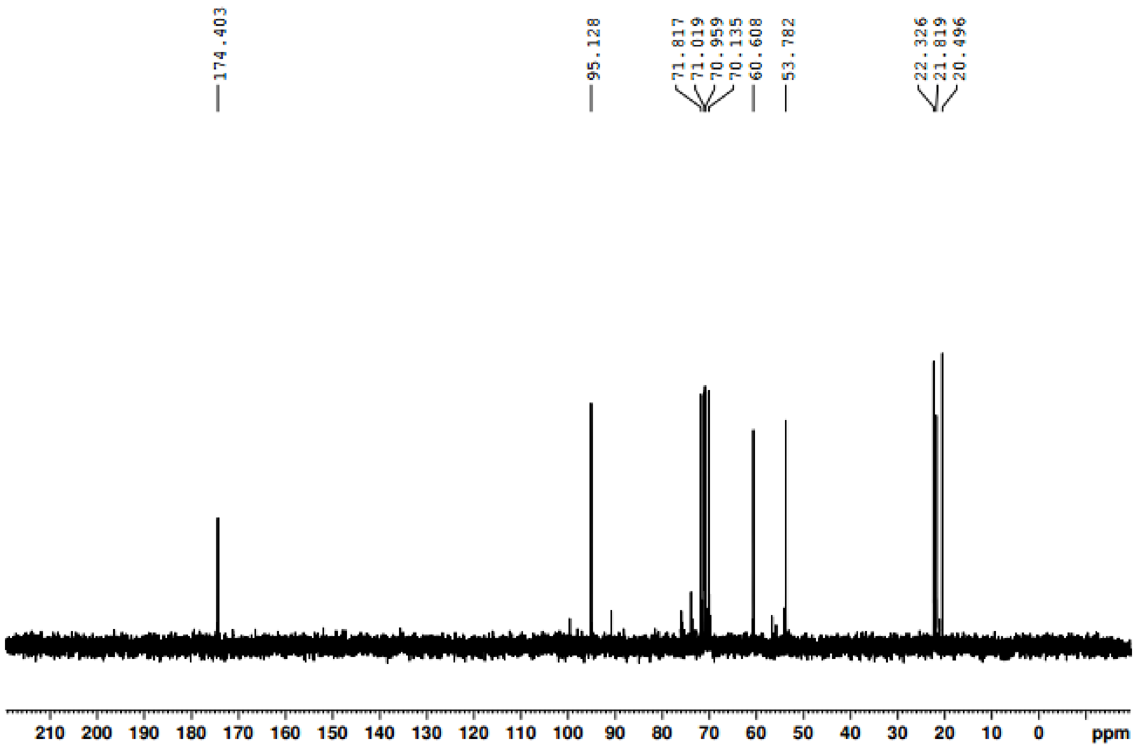

S1. I *N*-((3*R*,4*R*,5*S*,6*R*)-4,5-dihydroxy-6-(hydroxymethyl)-2-isopropoxytetrahydro-2*H*-pyran-3-yl) acetamide

The title compound was isolated by column chromatography (EtOAc/MeOH = 95:5) furnished compound **Glycoside-9** as white powder 0.11 g (46%); anomeric ratio (87:13). ^{1}H NMR (400 MHz, D_2O) δ 4.99 (d, *J* = 3.6 Hz, 1H, H-1β), 3.95-3.84 (m, 4H), 3.81-3.72 (m, 4H), 3.47 (t, *J* = 9.2 Hz, 1H), 2.05 (s, 3H), 1.22 (d, *J* = 6.4 Hz, 3H), 1.14 (d, *J* = 6 Hz, 3H); ^{13}C NMR (100 MHz, D_2O): δ 174.4 (C=O), 95.1 (C-1α), 71.8, 71.0, 70.95, 70.13, 60.60, 53.78, 22.32, 21.81,20.49; HRMS(ESI) *m/z* calcd for $C_{11}H_{21}NO_6$ $[M+Na]^+$ 286.1261, found 286.1030.

Disease Models & Mechanisms • Supplementary information

S1. J *N*-((3*R*,4*R*,5*S*,6*R*)-2-butoxy-4,5-dihydroxy-6-(hydroxymethyl)tetrahydro-2*H*-pyran-3-yl)acetamide

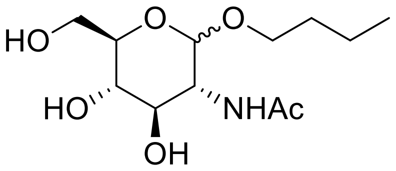

Glycoside-10

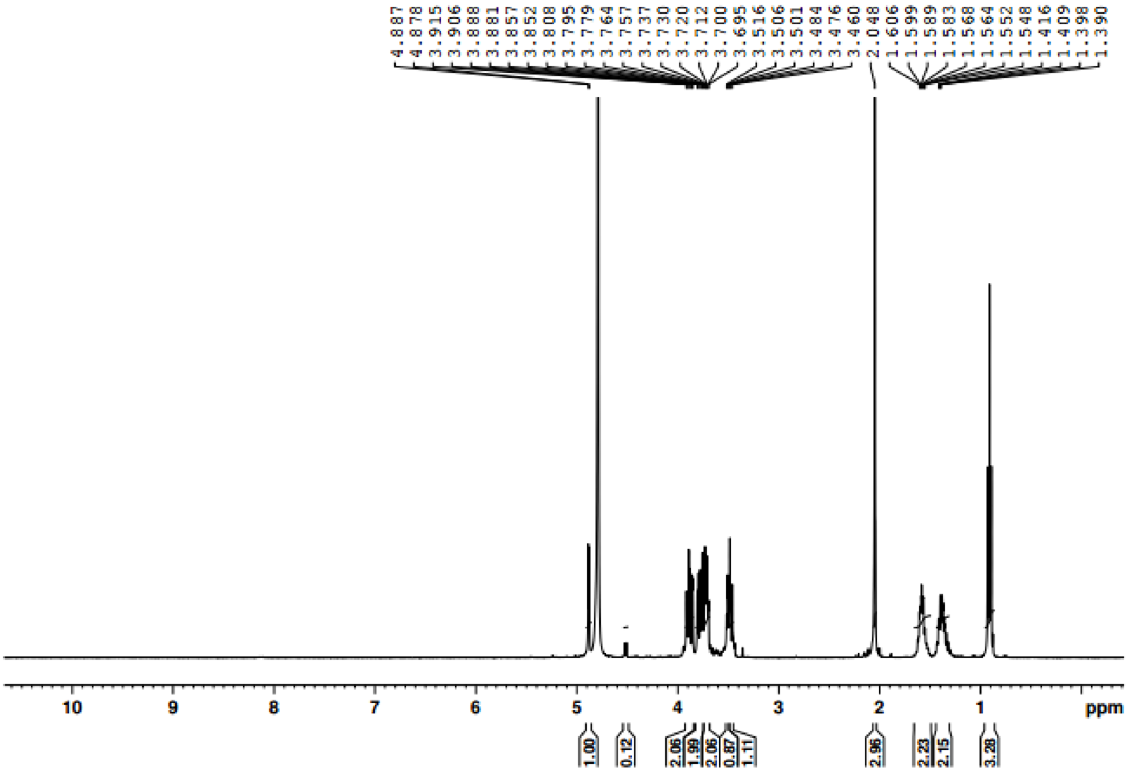

Disease Models & Mechanisms • Supplementary information

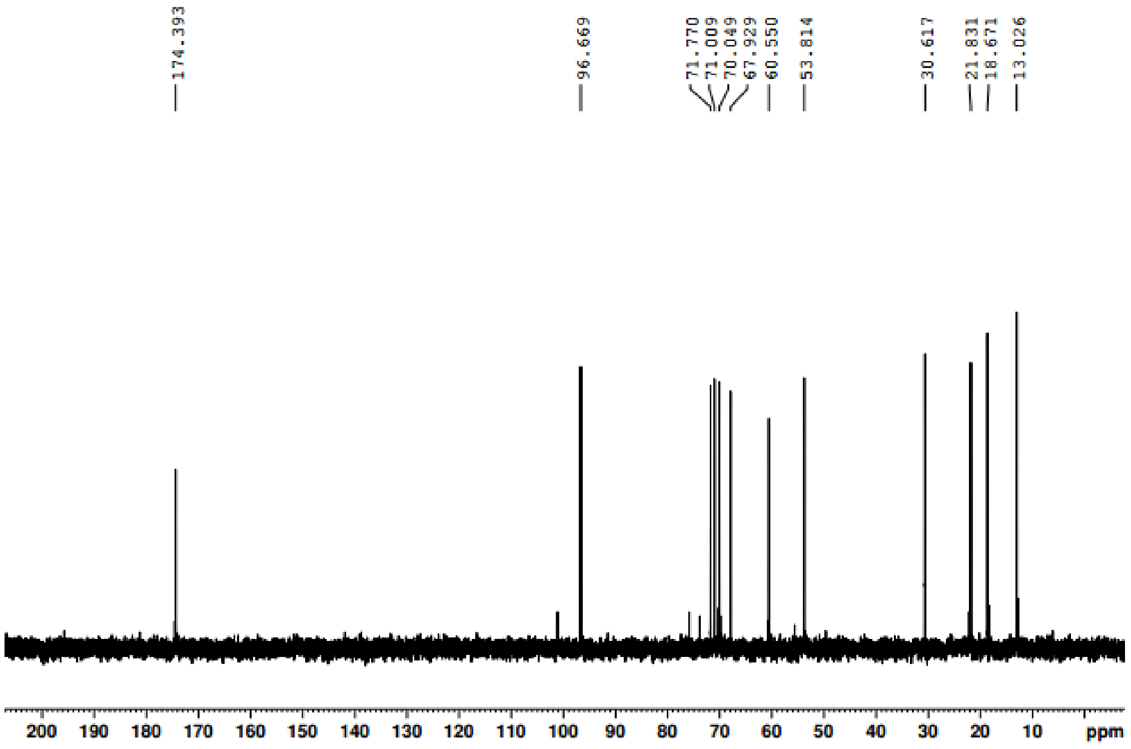

S1. J *N*-((3*R*,4*R*,5*S*,6*R*)-2-butoxy-4,5-dihydroxy-6-(hydroxymethyl)tetrahydro-2*H*-pyran-3-yl)acetamide

The title compound **Glycoside-10** was isolated by column chromatography (EtOAc/MeOH = 90:10) in 44% yield; anomeric ratio (α:β, 9:1).yellow color sticky liquid**;** ^{1}H NMR (400 MHz, D_2O): δ 4.87 (d, *J* = 3.6 Hz, 1H, H-1β), 4.51 (d, *J* = 8.4 Hz, 0.1H), 3.88 (td, *J* = 3.6 Hz, 10.8 Hz, 2H), 3.78 (dd, *J* = 5.2 Hz, 11.6 Hz, 2H), 3.75-3.69 (m, 2H), 3.51-3.46 (m, 2H), 2.04 (s, 3H), 1.6-1.54 (m, 2H), 1.41-1.35 (m, 2H), 0.91 (t, *J* = 7.2 Hz, 3H); ^{13}C NMR (100 MHz, D_2O): δ 174.3 (C=O), 101.1 (C-1α), 96.6 (C-1β), 71.7, 71.0, 70.0, 67.9, 60.5, 53.8, 30.6, 21.8, 18.6, 13.0**;** HRMS (ESI) *m/z* calcd for $C_{12}H_{23}NO_6$, $[M+Na]^+$ 300.1423, found 300.1427.

Disease Models & Mechanisms • Supplementary information

S1. K ***N*****-((3*****R*****,4*****R*****,5*****S*****,6*****R*****)-4,5-dihydroxy-6-(hydroxymethyl)-2-(isopentyloxy)tetrahydro-2*****H*****-pyran-3yl)acetamide.**

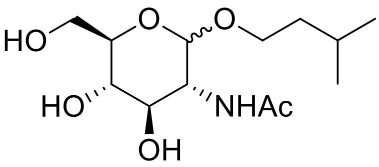

Glycoside-11

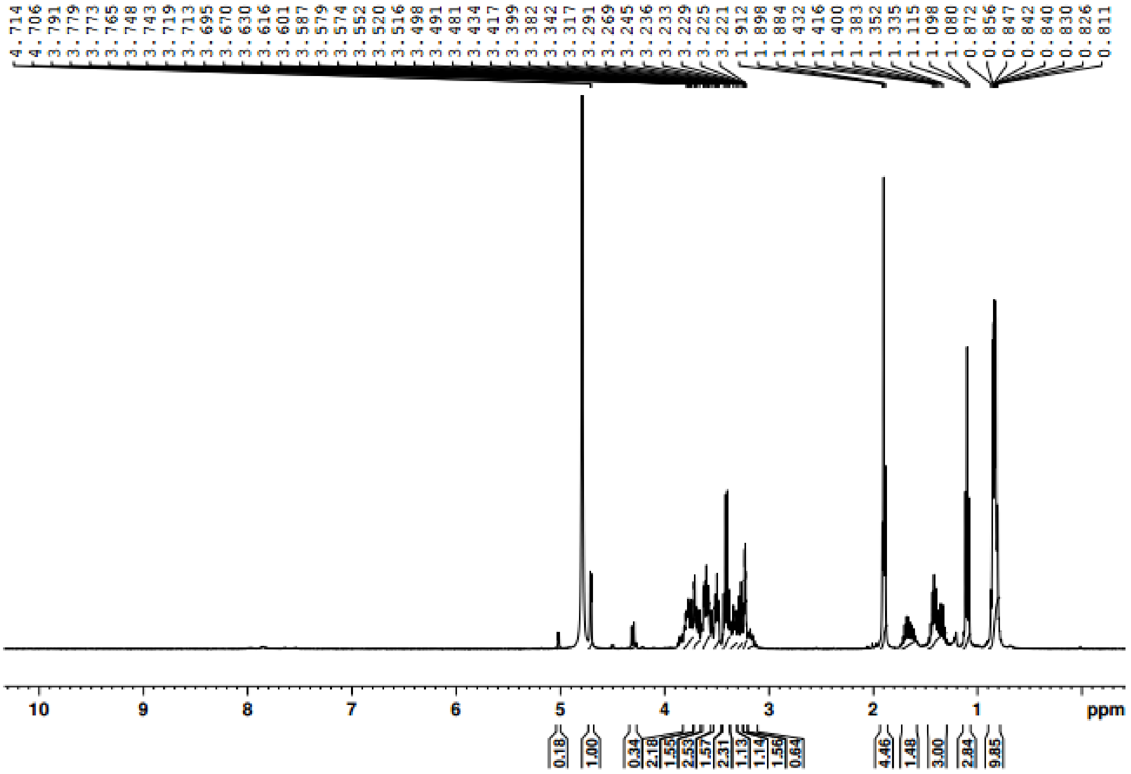

Disease Models & Mechanisms • Supplementary information

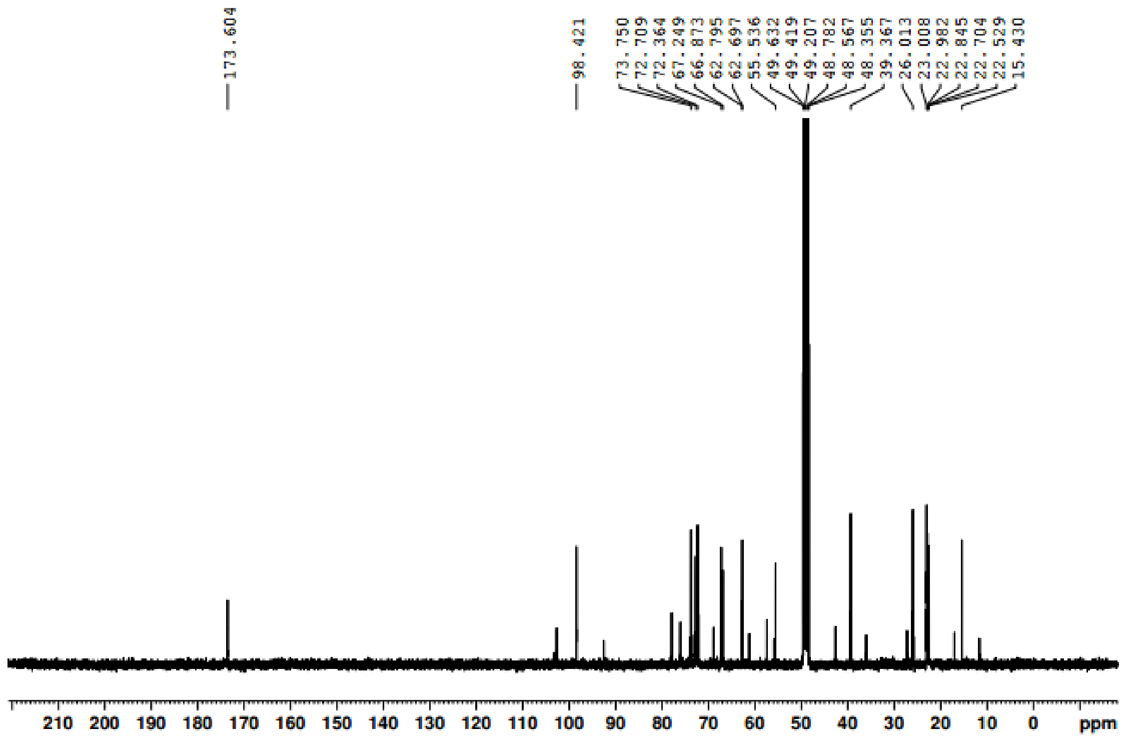

S1. K *N*-((3*R*,4*R*,5*S*,6*R*)-4,5-dihydroxy-6-(hydroxymethyl)-2-(isopentyloxy)tetrahydro-2*H*-pyran-3yl)acetamide.

The title compound **Glycoside-11** was isolated by column chromatography (EtOAc/MeOH = 95:5) yellow color sticky liquid in 44% yield, anomeric ratio (α : β, 85 : 15). ^{1}H NMR (400 MHz, CD_3OD): δ 4.78 (d, *J* = 3.2 Hz, 1H, H-1β), 4.38 (d, *J* = 8.4 Hz, H-1α), 3.89-3.84 (m, 1.4H), 3.83-3.75 (m, 2H), 3.71-3.65 (m, 3H), 3.60-3.56 (m, 1.5H), 3.49 (t, *J* = 7.6 Hz, 14 Hz, 2H), 3.41 (dd, *J* = 3.6 Hz, 6.4 Hz, 1H), 3.35 (dd, *J* = 3.6 Hz, 12.8 Hz, 1H), 3.30-3.21 (m, 0.5H), 1.97 (t, *J* = 5.2 Hz, 4H), 1.79-1.67 (m, 1H), 1.52-1.39 (m, 3H), 1.17 (t, *J* = 8 Hz, 2H), 0.93-0.89 (m, 10H); ^{13}C NMR (100 MHz, CD_3OD): δ 173.6 (C-1β), 102.7 (C-1α), 98.4, 92.5, 77.9, 76.0, 73.9, 73.0, 72.6, 72.4, 68.9, 62.7, 61.2, 57.3, 55.9, 55.6, 42.6, 39.4, 36.0, 27.1, 25.9, 25.7, 23.0, 22.9, 22.7, 22.6, 17.0, 11.6; HRMS (ESI) *m/z* calcd for $C_{13}H_{25}NO_6$, $[M+Na]^+$ 314.1574, found 314.1611.

Disease Models & Mechanisms • Supplementary information

S1. L *N*-((3*R*,4*R*,5*S*,6*R*)-2-(hexyloxy)-4,5-dihydroxy-6-(hydroxymethyl)tetrahydro-2*H*-pyran-3-yl)acetamide

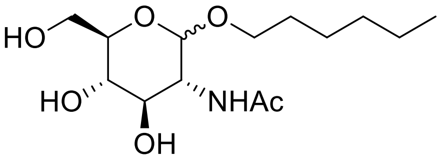

Glycoside-12

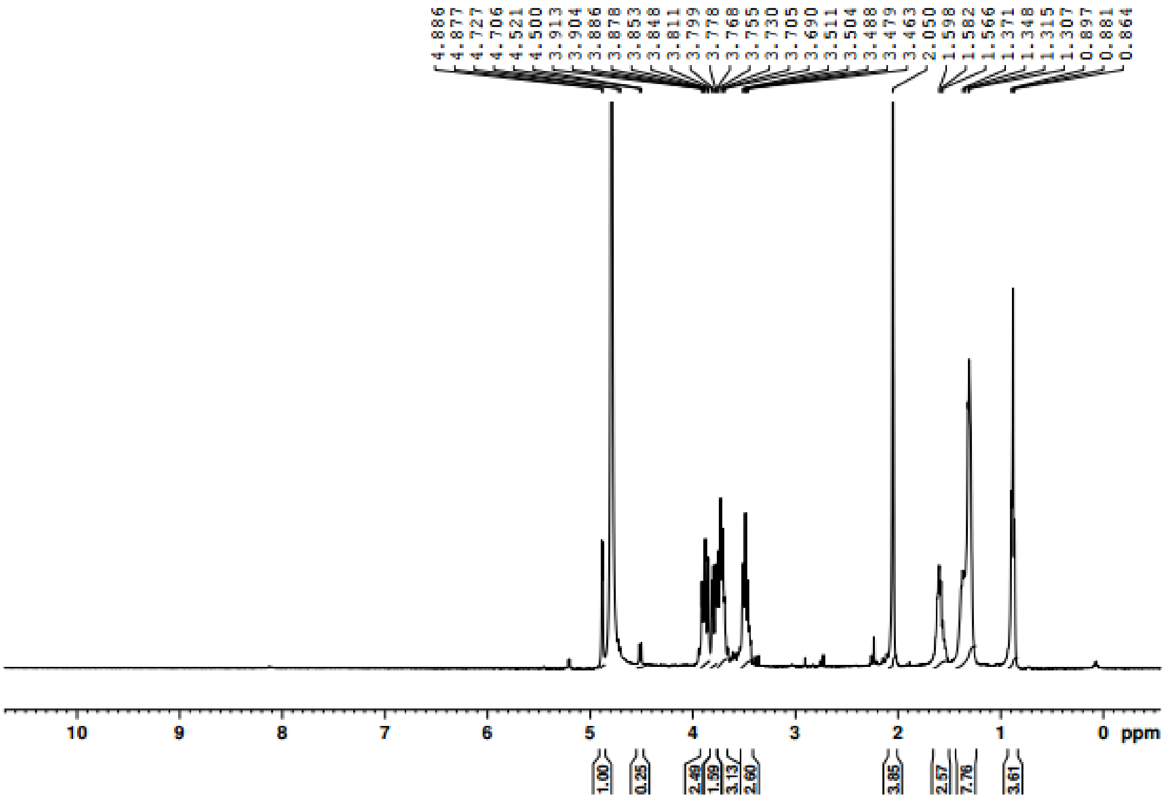

Disease Models & Mechanisms • Supplementary information

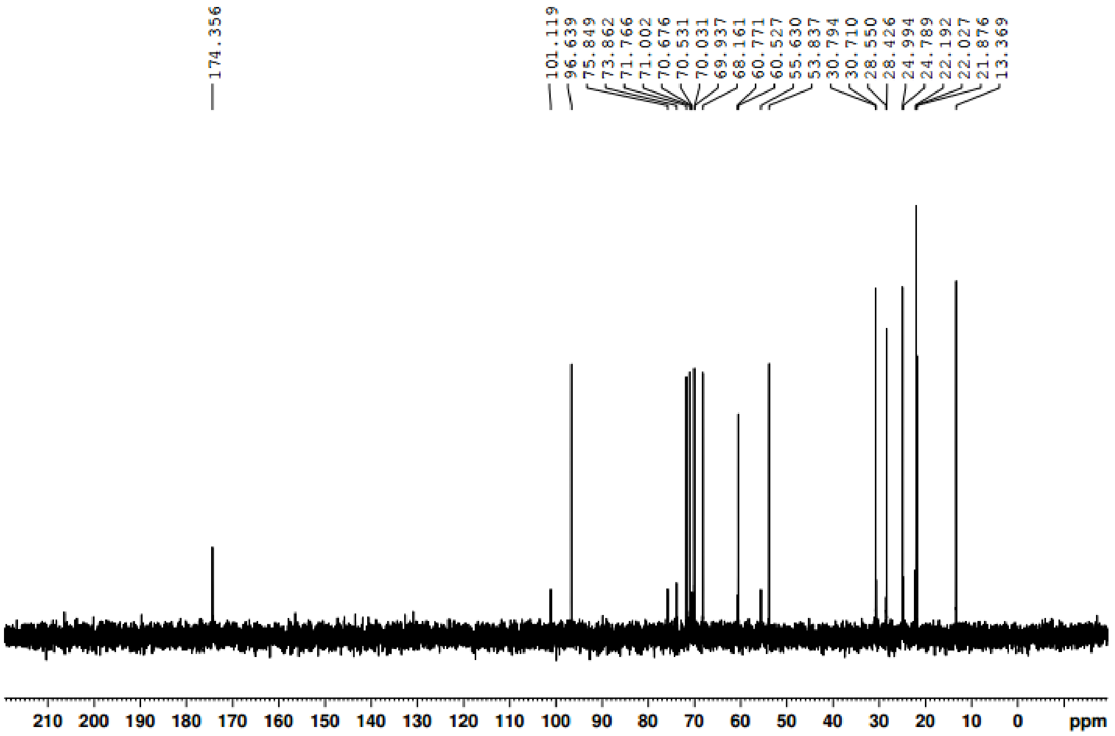

S1. L *N*-((3*R*,4*R*,5*S*,6*R*)-2-(hexyloxy)-4,5-dihydroxy-6-(hydroxymethyl)tetrahydro-2*H*-pyran-3-yl)acetamide

The title compound **Glycoside-12** was isolated by column chromatography (EtOAc/MeOH = 95:5) sticky solid compound 0.12 g (41%). anomeric ratio (α : β, 8:2); ^{1}HNMR (400 MHz, CD_3OD): δ 4.88 (d, *J* = 3.6 Hz, 1H, H-1β), 4.51 (d, *J* = 8.4 Hz, 1H, H-1α), 3.97-3.81 (m, 2H), 3.78 (dd, 2H), 3.75-3.69 (m, 3H), 3.51-3.46 (m, 2H), 2.05 (s, 3H), 1.58 (m, 2H), 1.37-1.30 (m, 7H), 0.88 (t, *J* = 6.4 Hz, 3H); ^{13}C NMR (100 MHz, D_2O): δ 174.3 (C=O), 101.1, 96.6 (C-1β),75.8, 73.8, 71.7, 71.0, 70.6, 70.5, 70.0, 69.9, 68.1, 60.7, 60.5, 55.6, 30.8, 30.7, 28.5, 28.4, 24.9, 24.7, 22.1, 21.8, 13.3 HRMS(ESI) *m/z* calcd for $C_{14}H_{27}NO_6$ $[M+H]^+$ 328.1731, found 328.1773.

Disease Models & Mechanisms • Supplementary information

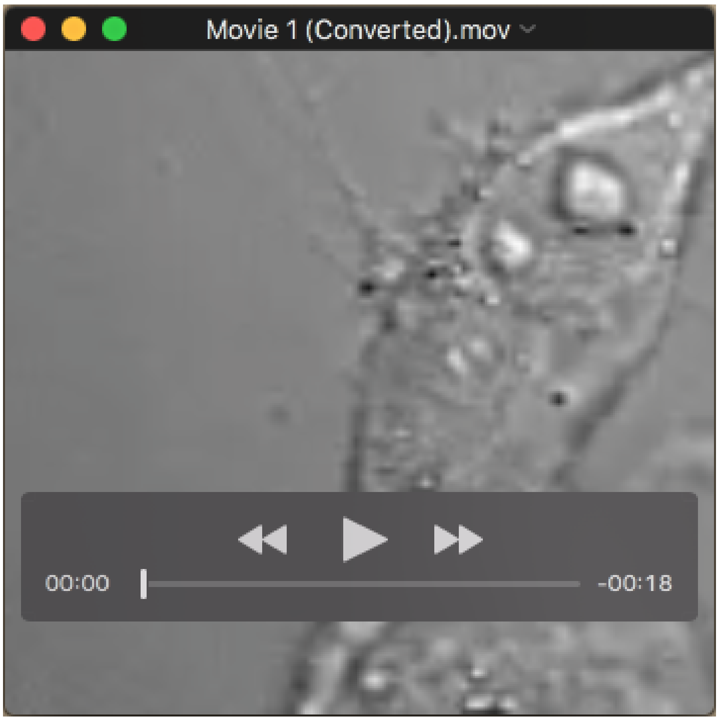

Movie 1: Time lapse video showing blebbing of MDCK in the presence Epsilon toxin

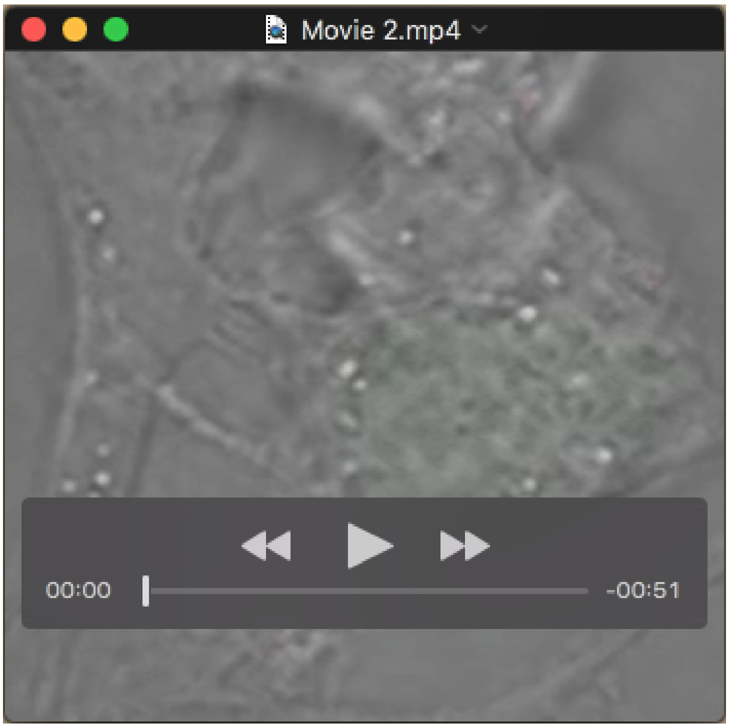

Movie 2: Time lapse video showing sequential increase in intracellular Calcium levels (Fluo-4 AM) and PI (Propidium Iodide) positivity in the presence of Epsilon toxin exposed MDCK

Disease Models & Mechanisms • Supplementary information
